# ANN-QSAR, Molecular Docking, ADMET Predictions, and Molecular Dynamics Studies of Isothiazole Derivatives to Design New and Selective Inhibitors of HCV Polymerase NS5B

**DOI:** 10.3390/ph17121712

**Published:** 2024-12-18

**Authors:** Maroua Fattouche, Salah Belaidi, Oussama Abchir, Walid Al-Shaar, Khaled Younes, Muneerah Mogren Al-Mogren, Samir Chtita, Fatima Soualmia, Majdi Hochlaf

**Affiliations:** 1 Group of Computational and Medicinal Chemistry, Molecular Chemistry and Environment Laboratory, University of Biskra, BP 145, Biskra 07000, Algeria; s.belaidi@univ-biskra.dz; 2 Laboratory of Analytical and Molecular Chemistry, Hassan II University of Casablanca, B.P, 7955 Casablanca, Morocco; oussamaabchir12@gmail.com (O.A.); samirchtita@gmail.com (S.C.); 3 College of Engineering and Technology, American University of the Middle East, Egaila 54200, Kuwait; walid.alshaar@aum.edu.kw (W.A.-S.); khaled.younes@aum.edu.kw (K.Y.); 4 Department of Chemistry, Faculty of Sciences, King Saud University, Riyadh 11451, Saudi Arabia; mmogren@ksu.edu.sa; 5 Process Engineering and Environment Laboratory, University of Science and Technology of Oran (USTO), BP 1503, Oran 31000, Algeria; soualmia-gc@outlook.fr; 6 COSYS/IMSE, Université Gustave Eiffel, Champs-sur-Marne, 77454 Marne-la-Vallée, Cedex 2, France; majdi.hochlaf@univ-eiffel.fr

**Keywords:** polymerase NS5B, isothiazole derivatives, ANN-QSAR, ADMET, molecular docking, molecular dynamics

## Abstract

**Background/Objectives:** RNA polymerase (NS5B), serves as a crucial target for pharmaceutical interventions aimed at combating the hepatitis C virus (HCV), which poses significant health challenges worldwide. The present research endeavors to explore and implement a variety of advanced molecular modeling techniques that aim to create and identify innovative and highly effective inhibitors that specifically target the RNA polymerase enzyme. **Methods:** In this study, a QSAR investigation was carried out on a set of thirty-eight isothiazole derivatives targeting NS5B inhibition and thus hepatitis C virus (HCV) treatment. The research methodology made use of various statistical techniques including multiple linear regression (MLR) and artificial neural networks (ANNs) to develop satisfactory models in terms of internal and external validation parameters, indicating their reliability in predicting the activity of new inhibitors. Accordingly, a series of potent NS5B inhibitors is designed, and their inhibitory potential is confirmed through molecular docking simulations. **Results:** These simulations showed that the interactions between these inhibitors and the active site 221 binding pocket of the NS5B protein are hydrophobic and hydrogen bond interactions, as well as carbon–hydrogen bonds and electrostatic interactions. Additionally, these newly formulated compounds displayed favorable ADMET characteristics, with molecular dynamics investigations revealing a stable energetic state and dynamic equilibrium. **Conclusions:** Our work highlights the importance of NS5B inhibition for the treatment of HCV.

## 1. Introduction

Hepatitis C virus (HCV) is classified in the Flaviviridae group and is identified as an RNA virus. This virus replicates itself using this genetic material without host cells. In 1997, World Health Organization (WHO) stated that this infectious agent poses a significant global health risk, impacting over 170 million individuals worldwide (i.e., ~3.3% of the total global population). The infection caused by HCV has the potential to progress to chronic hepatitis for ~70% of infected persons, with a wide range of uncertainty spanning from 50% to 85%, indicating the variability in disease progression among different individuals. In cases where an acute infection develops into chronic hepatitis, there is a notable 80% risk of having more or less serious complications, such as liver cirrhosis and hepatocarcinoma (a liver cancer). Among these hepatic complications, HCV infection is frequently documented, and it is unfortunate to discover that effective medications with minimal side effects are not currently available [1].

In the beginning of the 21st century, an HCV global epidemic infection emerged, most likely inadvertently transmitted during medical interventions. The increased utilization of parenteral therapies and blood transfusions played a significant role in fueling the epidemic. In developed countries, the implementation of strict health practices and the introduction of screening measures for the presence of antibodies (anti-HCV) in blood banks have led to a remarkable decrease in HCV infections. However, the epidemic continues to wreak havoc in developing countries, where the virus is transmitted in different ways such as blood transfusions, not properly screened, and the use of needles that are not properly sterilized for medical procedures. The administration of certain pharmaceutical drugs, as well as practices like injections and blood transfusions, has been linked to the occurrence of severe side effects in patients, thereby underscoring the need for safer and more cost-effective treatment options. Given the existing challenges and limitations in the current therapeutic approaches for HCV infection, there exists a pressing demand within the medical community for the development of novel anti-HCV agents that can complement and enhance the efficiency of existing treatments [2].

The determination of the activity of small molecule inhibitors in vitro is a process that consumes a considerable amount of time and resources, thereby incurring high costs. However, computer-aided drug design (CADD) techniques offer promising alternatives. CADD not only has the capability to predict the activity of inhibitors at an early stage but also has the potential to reduce experimental expenses significantly. Moreover, it can provide valuable insights to aid in the design of new inhibitors that may exhibit enhanced effectiveness. Additionally, CADD serves as a valuable tool for determining the reaction mechanisms of inhibitors at the molecular level, thereby contributing to a deeper understanding of their mode of action. Computational methodologies such as the quantitative structure–activity relationships (QSAR) approach plays a pivotal role in the design of novel drugs. By leveraging the best statistical models that incorporate the molecular descriptors, QSAR enables the prediction of various properties of these compounds, including their activity and potential toxicity. Various regression techniques and pattern recognition methods are utilized in the process of the variable selection and development of QSAR models. Also, molecular docking simulations are widely employed in the field of drug design to predict the conformation of ligands and their interactions with the active site of the receptors, which could be proteins or enzymes. Furthermore, computational absorption, distribution, metabolism, excretion, and toxicity (ADMET) analysis evaluates the oral bioavailability and potential toxicity of compounds [3]. At present, we use several such in silico methodologies aiming at formulating orally bioavailable, non-nucleoside inhibitors targeting the HCV RNA-dependent RNA polymerase inhibition. This polymerase is an essential enzyme involved in the replication of the virus within host cells. Therefore, we aim to contribute towards the advancement of therapeutic strategies for combating HCV infection and improving the clinical outcomes for individuals affected by this significant public health concern [4].

Recently, isothiazole derivatives have emerged as potent NS5B inhibitors. The inhibition of this protein is established as a key step towards HCV treatment. Various studies have been conducted employing different methodologies, including a contour map-based approach, to gain insights into the activity profiles of isothiazole derivatives. This method serves as a valuable tool for comprehending their characteristics and offers crucial information for the future design of new NS5B inhibitors. This work thus explores the potential of isothiazole derivatives given in Table 1, as NS5B inhibitors as proposed in Refs. [5,6].

We analyze the correlation between the chemical structure of these inhibitors and their biological activity as determined experimentally. We evaluate their molecular descriptors, as computed by quantum chemical methods, aligning with prior research efforts establishing correlations between electronic descriptors and bioactivity. Within this framework, four compounds are put forward, identifying them as promising NS5B inhibitors. Additionally, molecular docking is employed to identify the binding sites and conserved pockets for the active compounds against NS5B. Subsequently, the proposed new biocompounds are subjected to molecular dynamics analysis based on the lowest docking energy for confirmation of their stability within the active NS5B receptor. Predictions concerning the pharmacokinetic properties of new biocompounds are made through calculations of ADMET parameters [7]. In conclusion, the findings of this work are expected to be useful for the development of specific and powerful NS5B agonists.

## 2. Results and Discussion

### 2.1. QSAR Modeling

The process of QSAR modeling involves the utilization of seventeen molecular descriptors and the evaluation of the biological activity of thirty-eight isothiazole derivatives as inhibitors of HCV NS5B polymerase at the active site. The data obtained from this analysis are presented in Table 2. To identify the best designed correlation models among the selection of non-collinear descriptors, the multi-linear regression method is employed.

The final linear model, as shown in Equation (1), involves four molecular descriptors, namely MW, Log P, HBA, and E_LUMO_:

pIC_50_ = 3.20212 + 0.01151 × MW − 0.68167 × Log P − 0.69025 × HBA − 46.95968 × E_LUMO_ (1)

This equation provides the most accurate representation of the relationship between the selected descriptors and the inhibitory biological activity of the compounds. Indeed, the statistical parameters associated with the model are as follows: N = 30, R^2^ = 0.811, R^2^_adj_ = 0.781, R^2^_test_ = 0.891, F = 26.794, MSE = 0.420, and Q^2^_LOO_ = 0.737, with a *p*-value < 0.0001, where N represents the number of ligands that are used in the training set. The R^2^ value indicates that the designed model was able to explain 81.1% of the experimental variance, suggesting a better correlation between predicted data and experimental data. This is further supported by the low value of MSE, and the high values of R^2^_adj_ = 0.781 and R^2^_test_ = 0.891, documenting a good fit of the model to the data. The Fisher test, with an F value of 26.794 and a *p*-value well below 0.005, proves the statistical significance of the regression equation. Also, since the Q^2^_Loo_ value of 0.737 is > 0.5, this signifies the validity of the constructed QSAR model. Hence, we can state that the model designed a substantial amount of information regarding the relationship between the molecular descriptors and the inhibitory activity [8].

In order to assess the intercorrelation between the descriptors in the model, the statistics and Pearson’s correlation coefficients were calculated and reported in Table 3. The low values of correlation coefficients between the molecular descriptors indicate a remarkable absence of intercorrelation between the latter. This finding is further supported by the low value of the Variance Inflation Factor (VIF) [9], which measures the extent of collinearity between variables. Indeed, VIF values are < 4 for two descriptors and <8 for the descriptors MW and HBA, in line with the absence of collinearity between the chosen molecular descriptors. In sum, our model has good stability and reliability.

### 2.2. Y-Randomization Test

The stability, reliability, and robustness of the constructed QSAR model are thoroughly examined and validated using the Y-Randomization method. Indeed, numerous random rearrangements of the Y vector are meticulously used. Specifically, after conducting 101 random trials [9], 101 iterative iterations are conducted in order to reconstruct 101 linear models randomly. The values of R, R^2^, and Q^2^ for the new models are presented in Table S1 of the Supplementary Material, which shows that the average values obtained for R^2^, Q^2^, and cR^2^p are 0.139, −0.243, and 0.747, respectively. The latter value, which is significantly greater than the threshold of 0.5, unequivocally demonstrates the exceptional performance and reliability of the QSAR model. The empirical evidence presented in Table 4 further solidifies the validity of the aforementioned results, thereby solidifying the credibility and robustness of the constructed model.

**Figure 1:**
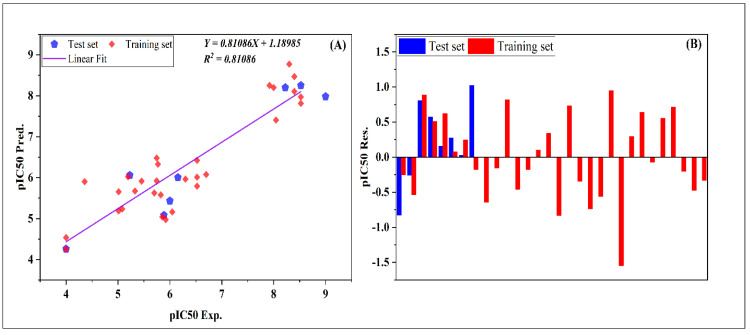
Plots of the predicted biological activity (pIC_50_ Pred.) versus the experimental one (pIC_50_ Exp.) and of the standardized residuals (pIC_50_ Res.) against the experimental activity of the substances under study are shown in (**A**) and (**B**), respectively.

The predicted activities of the test and training sets compared to the experimental results of pIC_50_ are presented in Figure 1A and Table 1. This figure highlights again the goodness of the established model, where one can see that the distribution of data is around the straight line of the graph. Additionally, the R^2^ values obtained from the plots align well with those from the test training sets and for both internal and external evaluations. In addition, the presence of any potential systematic error is explored. For instance, an examination of Figure 1B and Table 1, which show the experimental data of the pIC_50_ against the residual values of the training and test sets, shows that residuals spread on either side of zero, which indicates the absence of any systematic error. These results further confirm the robustness and precision of this QSAR model [10].

### 2.3. The Standards of Golbraikh and Tropsha

Golbraikh and Tropsha [11] proved the insufficiency of internal validation approaches to confirm the reliability and robustness of QSAR models. Consequently, external validations are crucial in order to build a dependable QSAR model [12]. In this study, a test set comprising eight compounds was reserved for external validation purposes as specified in Table 1. Table 5 gives the biostatistical parameters of the constructed model, as well as the r^2^m matrix. As can be seen there, the present parameters for our model fully satisfy the acceptability criteria established by Golbraikh and Tropsha.

### 2.4. Artificial Neural Networks (ANNs)

**Figure 2:**
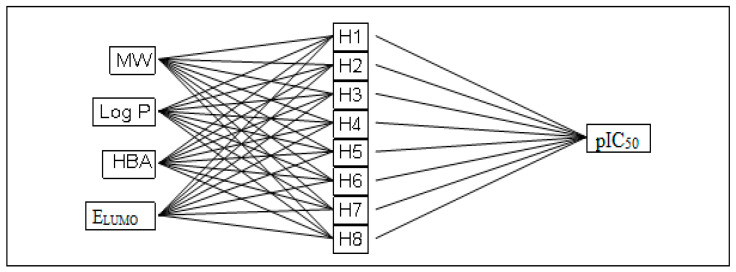
Structure of an ANN approach.

ANNs are computational models that are non-linear and prove to be quite valuable in predicting the biological activity of extensive datasets of molecules. Unlike traditional statistical techniques such as MLR or PLS, ANNs allow for the exploration of intricate and non-linear connections. Due to this characteristic, neural networks are exceptionally well-suited for applications in drug design and QSAR studies. They find broad utility in simulating various non-linear complex systems within domains such as pharmaceuticals, engineering, psychology, and medicinal chemistry. An example of the successful application of ANN is in the prediction and synthesis of novel organic chemical compounds. At present, an ANN model is constructed with four inputs representing descriptors chosen from the correlation matrix, eight hidden neurons, and one output neuron representing pIC_50_ (Figure 2) [13].

**Figure 3:**
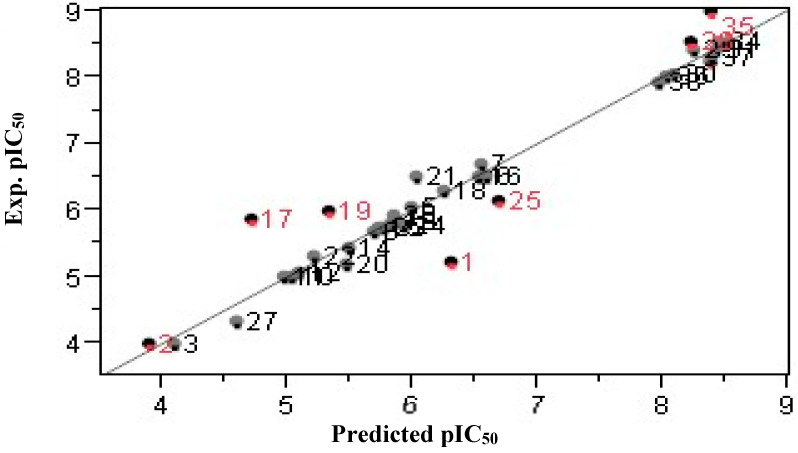
Correlation between experimental and predicted pIC_50_ estimated using ANN. The black (red) dots correspond to compounds of the training (test) set.

After adjustments and benchmarks, eight neurons in the hidden layer led to the highest correlation between predicted and experimental values. Subsequently, the ANN technology was based on the Gauss–Newton method, which resulted in a strong correlation between predicted and experimental pIC_50_ values (recorded in Table 6). This correlation is graphically depicted in Figure 3, where the R^2^, coefficient of variation (CV), and R^2^_test_ values were calculated to be R^2^ = 0.991, R^2^_cv_ = 0.939, and R^2^_test_ = 0.839, respectively. An analysis of the results from both the training and test sets (Figure 3) reveals that the ANN model with a (4-8-1) architecture effectively establishes a reliable relationship between the four descriptors and the anti-HCV activity of the isothiazoles derivatives under investigation. Notably, all test molecules (1, 2, 17, 19, 25, 28, 32, and 35) exhibit strong alignment with the ANN model, indicating its robustness [14].

### 2.5. The Applicability Domain Approach

**Figure 4:**
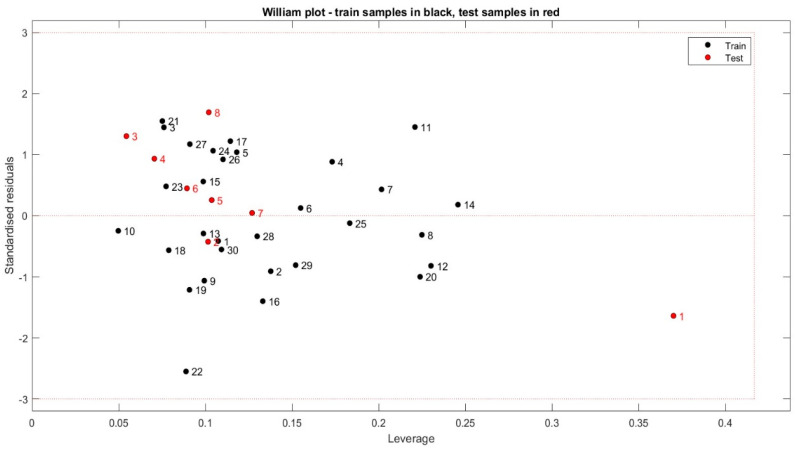
Application field of the Williams plot for the QSAR model.

To further evaluate the model, a plot of standardized residuals versus leverages, also known as the Williams plot, is generated and depicted in Figure 4. This plot shows that all compounds in the test and training sets are in the applicability domain since their leverage values are lower than the warning leverage (h* = 0.5 (h* = 3 × (k + 1)/n) where k = 4, N = 30) and their standardized residuals are falling within the range of ±3. This observation suggests the absence of outliers or influential compounds, thus allowing for the use of any compound in the design of new potent isothiazole derivatives.

### 2.6. Design and Selection of New Isothiazole Derivatives

The constructed QSAR model is used as a reference to designing new isothiazole derivatives targeting selective inhibition of HCV Polymerase NS5B. These newly designed compounds (denoted as Ni, with i = 1–10, Table 7) exhibit a predicted pIC_50_ values which are higher than that of the most active synthesized molecule (i.e., compound **35**) having a pIC_50_ ~7.9 (Table 1). For instance, Table 7 shows the details of the newly designed compounds, including their chemical structures, predicted pIC_50_ activity data, descriptors, binding energy, and leverage effects (h), while their corresponding smiles are available in Table S2 of the Supplementary Material. Furthermore, Table 7 provides a comprehensive overview of the properties and characteristics of these newly designed compounds, allowing for further analysis and evaluation.

### 2.7. Docking Results

Molecular docking has gained significant traction as an approach for developing novel pharmaceuticals, primarily attributed to the time and financial expenses associated with in silico drug screening when compared to traditional in vitro experiments. For docking, we used the AutoDock 4.2.6 software package [15], where the four specifically designed compounds are docked to the target protein binding site’s 2IJN crystal structure by conducting the docking processes through the re-docking of the co-crystallized complex **221** into the NS5B enzyme. The RMSD for the co-crystallized bioligand is 0.679 Å, i.e., smaller than the acceptable value (2 Å). In fact, each ligand yielded a total of ten conformations, and the binding affinity of these proposed ligands with the 2IJN receptor is explicitly detailed in Table 8.

The computed docking scores for all designed compounds fell within the range of −8 to −9.3 kcal/mol, showcasing larger binding energies in comparison to the co-crystallized ligand **221**, which exhibited a score of −7.5 kcal/mol. The selection of compounds with the most optimal conformation was based on the lowest AutoDock score and the most favorable interactions. Furthermore, a comprehensive analysis was conducted to collectively examine the binding interactions along with their 2D and 3D correlations, as presented in Table 8. In addition, we visualized the binding sites of **N7**, **N8**, **N9**, **N10**, and the co-crystallized ligand **221** compounds utilizing the Discovery Studio software 2021 platform, highlighting various interactions such as hydrophobic interactions, electrostatic interactions, hydrogen bonds, and halogen bonds, as detailed in Table 8 and Table S3. For instance, Compound **N7** forms a total of nine conventional hydrogen bonds with oxygen and nitrogen atoms interacting with Arg158 (2.52 Å), Arg158 (2.03 Å), Arg158 (2.44 Å), Asn291 (2.32 Å), Asn291 (2.24 Å), Ser556 (2.50 Å), Gly557 (2.68 Å), Gly449 (2.53 Å), Asp318 (2.67 Å), in addition to a carbon–hydrogen bond with the amino acid of Asp318 (3.53Å). Furthermore, three attractive charge interactions are identified with Glu143 (5.50 Å) and Pi-Cation interactions with Lys141 (4.14 Å) and Arg158 (4.53 Å). This compound also exhibits hydrophobic interactions with Ser55 (3.71 Å and 4.26 Å) such as Pi-Sigma and amide-Pi stacking interactions. For Compound **N8**, we found five hydrogen bonds, including two bonds with the amino acid Arg394 and three others with Asn411, Val405, and Glu143. Furthermore, we identified three carbon–hydrogen bonds with the amino acid Ser407, each at distances of 3.65 Å, 3.54 Å, and 3.15 Å. Compound **N8** also exhibited an electrostatic band with the amino acid Glu398 at a distance of 2.60 Å and a salt bridge/attractive charge interaction type as well as another type of interaction: Pi–sulfur with Tyr415 at 5.70 Å. The lengths of the H-bonds are ~2.3–2.5 Å. For Compound **N9**, it is engaged in four H-bonds with Tyr415 (2.59 Å), Tyr448 (2.60 Å), Ser556 (2.39 Å), and Asp318 (2.30 Å). Additionally, it forms a Pi–Donor H-bond interaction with Ser556 and two hydrophobic interactions with the alkyl group of Met414 (4.45 Å), and a Pi–alkyl interaction with Cys366 (4.05 Å). Similarly, Compound **N10** participates in three H-bonds with amino acids Asn291 (2.27 Å), Asp318 (2.49 Å), and Asp318 (3.36 Å), along with a carbon–hydrogen bond with Asn316 (3.44 Å), together with electrostatic interactions (two attractive charge interactions with Asp225 (4.68 Å) and Asp318 (3.98 Å) and Pi–anion-type interaction with Asp318 (3.37 Å)). It is also engaged in two hydrophobic Pi–alkyl-type interactions with Val52 (5.16 Å), Arg158 (5.23 Å), and Cys223 (5.19 Å), and a Pi–sulfur interaction with Phe193 (5.85 Å). In sum, these weakly bound interactions participate in the stabilization of these inhibitors at the active site of NS5B.

The co-crystallized ligand **221** forms three traditional hydrogen bonds, with one nitrogen atom forming bonds with two amino acids, Tyr448 (2.52 Å) and Gly449 (2.19 Å), and one bond with a sulfur atom and Asn411 (3.74 Å). Additionally, it is engaged in three H-bonds involving the fluorine atom with three amino acids (namely, Asn316 (2.88 Å), Cys366 (2.86 Å), and Tyr415 (2.63 Å)). Hydrophobic interactions with three alkyl groups (Cys366 (4.38 Å), Leu384 (5.14 Å), Met414 (4.64 Å)) and five Pi–alkyl interactions with Phe193 (5.20 Å), Tyr415 (5.16 Å), Tyr448 (4.88 Å), Cys366 (5.14 Å), and Met414 (5.40 Å) participate to in the binding of this ligand with NS5B. In sum, the newly predicted inhibitors exhibit superior binding affinity, interactions, and stability compared to the ligand **221**. These findings suggest that the mentioned ligands are more effective inhibitors of NS5B due to their ability to form strong and stables complexes with the target protein [16] as confirmed by their larger, in absolute values, binding energies (see above).

### 2.8. ADMET Investigation and Drug-likeness

Drug-likeness predictions are performed based on Lipinski, Veber, and Ghose rules. The Lipinski filter [17] outlines the criteria for molecule absorption or permeation, stating that an MW under 500 g/mol, a log *p* value < 5, and a maximum of 5 H-donor and 10 H-acceptor atoms enhance the likelihood. The Ghose rule [18] defines drug-likeness parameters such as a MW between 160 and 480, a log P between −0.4 and 5.6, a total number of atoms between 20 and 70, and a MR between 40 and 130. The Veber filter [19] defines the drug-likeness parameters such as PSA ≤ 140 and the number of rotating bonds (n ≤ 10). The majority of the newly predicted inhibitors fulfill Lipinski, Veber, and Ghose criteria (Table 9). Moreover, synthetic accessibility scores indicate the ease of laboratory synthesis on a scale of 0 to 10, with all analogs having a score below 4.5, suggesting that they may have easy industrial synthesis. Thus, the newly developed compounds are deemed as highly potent anti-HCV agents.

The most crucial and challenging stage in the ongoing drug design and development involves conducting studies on drug metabolism and pharmacokinetics (DMPK), commonly known as ADME [20]. ADME studies provide insights into the physicochemical properties and drug-likeness predictions of the proposed compounds (**N7**, **N8**, **N9**, and **N10**) and of the co-crystallized compound **221**. Presently, ADME properties are assessed using Swiss ADME. Additionally, toxicity assessments are performed by the pkCSM approach. These data are depicted in Table 9.

The consideration of a compound’s aqueous and non-aqueous solubility is vital in the drug development process. An analysis of ADME results reveals that compounds **N7**, **N8**, **N9**, **N10** and **221** present a positive tolerance regarding the laws of the blood–brain barrier (BBB), indicating their ability to cross the BBB. Also, skin permeability, determined by the logarithm of Kp (log Kp), shows that all these molecules have the lowest skin permeability.

P-glycoprotein, also called Permeability-GlycoProtein or P-gp, which causes a phenomenon of multi-drug resistance, is mainly found in cells of the proximal convoluted tubule of the kidney, cells of the intestine, trophoblasts of the placenta, and the endothelium of the blood–brain barrier. P-glycoprotein acts as a biological defense mechanism by expelling toxins and xenobiotics from cells [21]. Compounds **N7** and **N8** are identified as substrates for P-glycoprotein, whereas **N9**, **N10**, and **221** are not. In addition, the impact of cytochrome P450 metabolism, particularly CYP3A4, is significant in humans. Compounds **N7**, **N8,** and **N10** are found to be inhibitors of CYP3A4 but not of CYP1A2, CYP2C19, CYP2C9, or CYP2D6. In contrast, the co-crystalline compound **221** inhibits CYP1A2 and CYP2C19. Toxicity assessments indicate that the newly developed compounds **N7**, **N8**, **N10**, and **221** are expected to be non-mutagenic, do not induce skin sensitization and exhibit no HEGR inhibition. In summary, these compounds have favorable ADMET profiles and pharmacological similarities.

### 2.9. Dynamics Simulation

**Figure 5:**
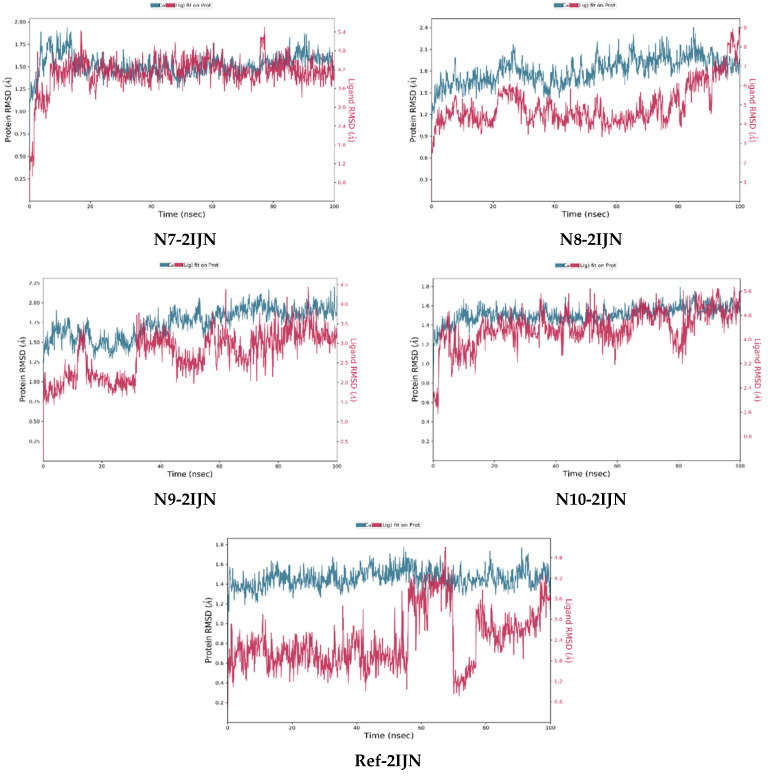
RMSD plots of simulated complexes (**N7**, **N8**, **N9**, **N10**, and reference drug (**221**) complexed with **2IJN** receptor).

**Figure 6:**
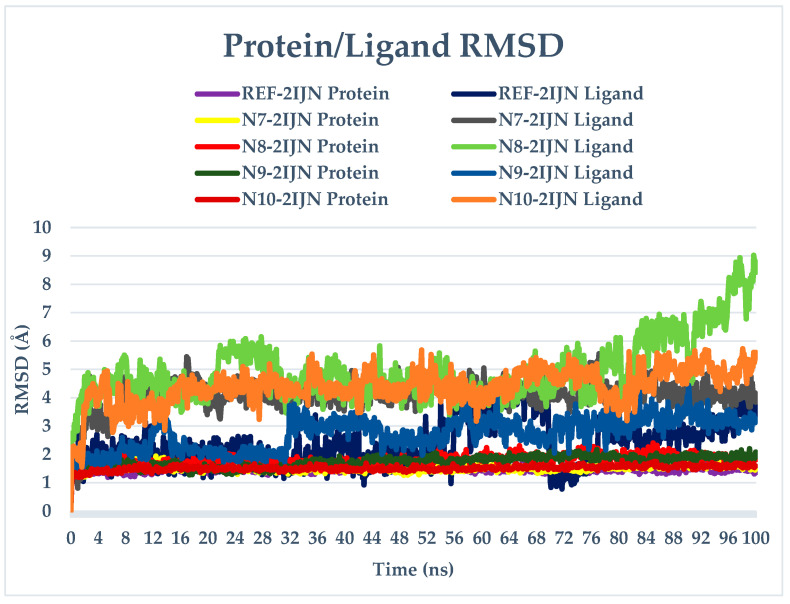
RMSD plots of singular graphical of proteins/ligands.

**Figure 7:**
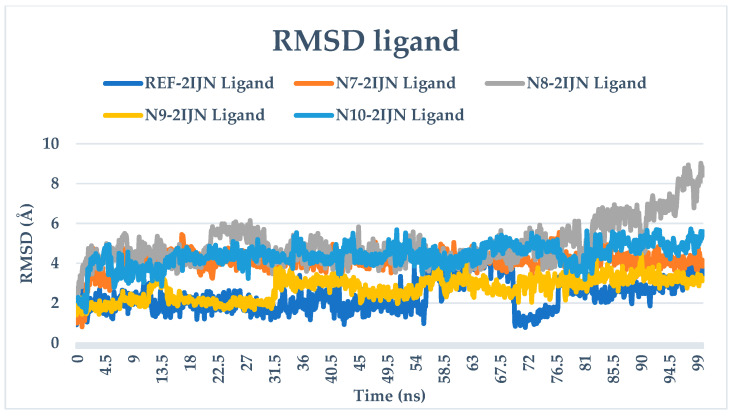
RMSD plots of singular graphical of ligands.

**Figure 8:**
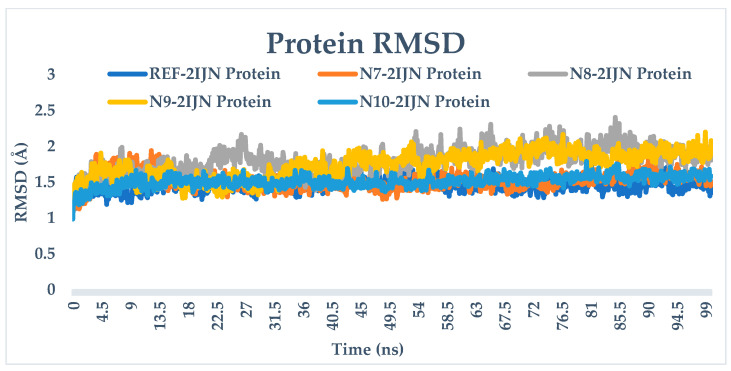
RMSD plots of singular graphical of protein.

The RMSD analysis provides valuable insights into the stability of both the protein and ligands during the simulation. The RMSD values for all complexes, including protein unbound ligands **N7**, **N8**, **N9**, and **N10**, as well as the reference drug, are calculated and depicted in Figure 5. The RMSD analysis reveals that the protein structures remain relatively stable throughout the entire simulation, with deviations not exceeding 3 Å. This suggests that only minor changes occur in the protein structure over the course of all simulations. The distinct Root Mean Square Deviation (RMSD) profiles that pertain to each individual system under investigation were meticulously superimposed onto a singular graphical representation, wherein a diverse array of colors was employed to distinctly differentiate and visually categorize each profile in the corresponding Figure 6, Figure 7 and Figure 8, which are presented, respectively, for clarity and ease of analysis.

For compounds **N7**, **N8,** and **N10**, they display consistent and stable RMSD patterns, indicating minimal structural variations during the simulation period. In contrast, the reference drug and compound **N9** exhibit noteworthy fluctuations in their RMSD values. This suggests that these specific ligands undergo more significant structural changes during the simulation compared to the other compounds. Therefore, the slight changes observed in the protein structure and the stability of most ligands imply that the overall system remains relatively stable. However, the distinct fluctuations in the RMSD values of the reference drug and compound **N9** warrant further investigation into the specific molecular dynamics and interactions driving these variations.

**Figure 9:**
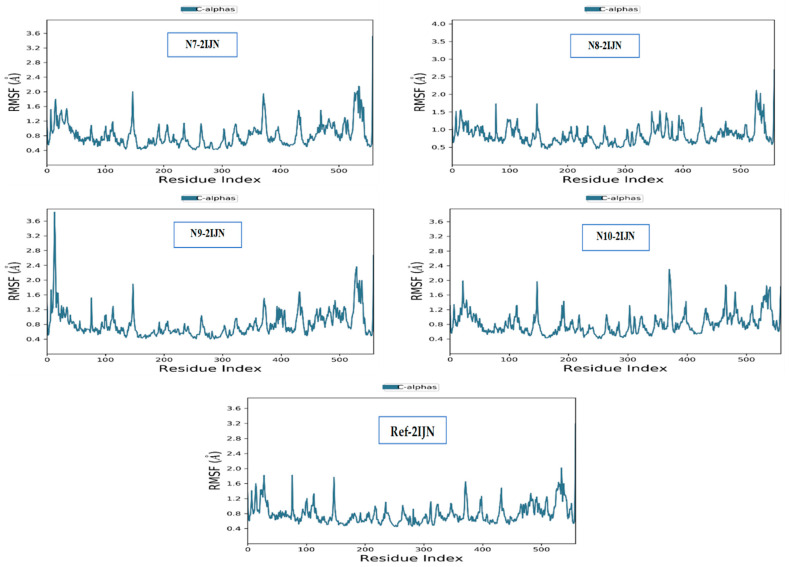
RMSF plots of proteins when complexed with the newly designed efficient inhibitors, and with reference drug (**REF-2IJN**).

The RMSD analysis and the RMSF plot are calculated for all the proteins in the complexes (Figure 9). The RMSF plot reveals some subtle fluctuations across all residues for each complex. Importantly, the RMSF values for all residues remained below the 3Å threshold, indicating a consistent stability in the overall protein structure throughout the simulations. Thus, this further corroborates the findings from the RMSD analysis, thus establishing the stability of the inhibitor–protein complexes. The slight fluctuations observed in the RMSF values suggest that the individual residues within the proteins experience minimal deviations from their average positions, reinforcing the notion of a structurally stable protein conformation during the simulation.

**Figure 10:**
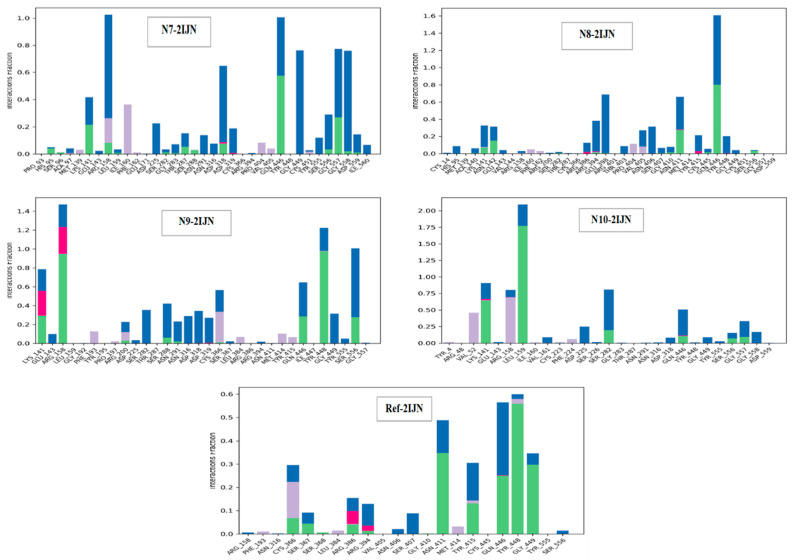
Protein–ligand contact of simulated complexes (green: hydrogen bonds; pink: Ionic bonds; blue: water bridge; violet: hydrophobic bonds).

Figure 10 illustrates a multitude of interactions between the proteins and ligands in the studied complexes. Specifically, the reference drug exhibits a diverse set of interactions, forming hydrogen bonds with Asn411, Tyr15, Gln446, Tyr448, and Tyr449, ionic bonds with Arg386 and Arg394, hydrophobic bonds with Cys366 and Met414, and water bridges with Cys366, Ser407, Asn411, Tyr415, and Gln446. In the case of ligand **N7** interacting with **2IJN** protein, hydrophobic interactions are observed with Arg158 and Ile160, while H-bonds are formed with Lys141, Gln446, and Gly557, and ionic interactions with Asp318 and Asp319. Water bridges are also noted with Arg158, Asp318, Gln446, Gly449, Gly557, and Gly558. Compound **N8** engages hydrophobic interactions with Pro404 and Val405 of this protein, forms hydrogen bonds with Asn411 and Gln446, ionic bonds with Arg386 and Tyr415, and water bridges with Arg394, Glu398, Asn411, and Gln446. While complexed with 2IJN protein, Compound **N9** establishes hydrophobic interactions with Phe193, Arg200, Cys366, and Met414; H-bonds with Lys141, Arg158, Gln446, Tyr448, and Ser556; ionic interactions involving Lys141 and Arg158; and water bridges with Lys141, Arg158, Gln446, Ser556, Gly449, and Ser288. In the case of compound **N10** interacting with the active site of **2IJN** protein, hydrophobic interactions with Val52 and Arg158, H-bonds with Lys141, Leu159 and Ser282, ionic interactions involving Lys141 and Gln446, and water bridges with Leu159, Asp225, Gln446, Gly557, and Gly558 are observed. Noteworthy, these above-mentioned interactions are various and they correspond to specific molecular contacts between the inhibitors and the protein. Additionally, we would like to point out the central role played by certain enzyme residues, such as Gln446 and Lys141. This is a sign of their significance in stabilizing the inhibitors via the formations of several interactions across multiple ligands. These findings provide an understanding, at the molecular level, of the mechanisms governing the binding interactions within the newly designed inhibitor–2IJN protein complexes.

## 3. Methods and Materials

### 3.1. Information and Data Collection

Building upon the findings of Refs. [5,6], we gathered a total of 38 molecules that belong to the isothiazole derivatives family and possess inhibitory properties against HCV Polymerase NS5B. We converted the HCV inhibitory activities of the evaluated compounds, which were measured in IC_50_ (μM), to pIC_50_, where pIC_50_ = −log (IC_50_ × 10^−6^ [22]. To ensure the accuracy and reliability of our data analysis, we assessed the performance of our model by randomly selecting 6 compounds from the total pool of 38 molecules to form the test set (marked by * in Table 1), while the remaining 32 compounds were designated as the training set for the construction of the model. The molecular structures of the isothiazole derivatives were visualized using the ChemDraw 19.0 software program [23]. The equilibrium structures of these compounds (as shown in Table 1) were obtained using the B3LYP/6-31G(d,p) method. The geometry of the isothiazole subunit is planar. In fact, all dihedral angles are either ~0° or ~180°. These optimized structures are shown in Table S4 of the Supplementary Material. Table 1 also provides the measured pIC50 values, predicted pIC50 data and corresponding residual values.

### 3.2. Molecular Descriptors

A suitable methodology for determining the equilibrium structures of isothiazole derivatives is selected after benchmark calculations on the subunit of the series studied as discussed in Refs. [13,24]. Accordingly, the geometry optimizations of isothiazole derivatives were carried out using the PM3 method as implemented in the HyperChem 8.06 software package [25]. Subsequently, we re-optimized the structures using the DFT (density functional theory) approach, employing the Becke, 3-parameter, Lee–Yang–Parr (B3LYP) functional [9], where the atoms are described using the 6-31G (d,p) basis set. These computations were performed using the GAUSSIAN 09 software program [26]. Afterwards, we used the QSAR properties module available in the HyperChem (8.06) and Marvin Sketch software packages 2019 [27] to evaluate the molecular descriptors of the isothiazole derivatives (Table 2). The latter include the partition coefficient (Log P), molar refractivity (MR), molar volume (MV), molar polarizability (Pol), surface area grid (SAG), molar weight (MW), polar surface area (PSA), distribution coefficient (Log D), hydration energy (HE), hydrogen bond donors (HBDs), hydrogen bond acceptors (HBAs), density (D), and number of rotatable bonds (NRB). Additionally, we employed the GAUSSIAN 09 software package to compute the dipole moment (μ), which is a measure of the charge distribution within the molecule. We also calculated the HOMO (highest occupied molecular orbital energy) and the LUMO (lowest unoccupied molecular orbital energy). We thus deduced the gap, which is the energy required to promote an electron from the HOMO to the LUMO. Finally, we computed the total energy (ET) of the molecules, which provides information about their overall stability and reactivity.

### 3.3. QSAR Studies

- Pretreatment and division of the dataset

In order to create a robust model, we conducted thorough processing of the previously calculated values of the molecular descriptors using the method proposed by Apablaza et al. [28], which involves the removal of correlated and repetitive values with a large percentage. For those purposes, we utilized the XLSTAT software version 2014 [29]. As part of this methodology, we randomly divided the group of treated descriptors into two subsets, 80% as the training group and 20% as the test group, thus ensuring a balanced representation of the data.

- Model development

Multiple linear regression (MLR): To obtain the regression of all subgroups within the training group, we employed the MLR method. MLR helps in building a correlation between the independent and dependent variables. Here, the dependent variable is the biological activity and the independent variables are molecular descriptors obtained from calculations, ensuring reproducibility and simplicity of its operation. Furthermore, MLR is a commonly used method in QSAR due to its simplicity, ease of interpretation, and analysis of the features applied for linear regression analysis. Its transparent nature makes it highly regarded in the field, as it facilitates the implementation of the algorithm and the application of predictions [30]. As part of this analysis, we aimed to eliminate any abnormal descriptors, resulting in the following equation:

(2) $$Y=a_{0}+i=1naixi$$

where *Y* represents the studied activity. *a*_0_ is the intercept of the equation. *x_i_* and *a_i_* represent the molecular descriptors and their coefficients [30].

Artificial neural network (ANN): This method takes advantage of computer models based on artificial intelligence. An ANN is a group of interconnected nodes, inspired by a simplification of the neurons in a brain. This method involves establishing a correlation between descriptors obtained from MLR models and the corresponding biological activities they exhibit. Within ANN architecture, there are three crucial layers that play distinct roles: the input layer, which accommodates a number of neurons equal to the number of descriptors deduced from the MLR model; the hidden layer, where complex patterns and relationships are identified; and the output layer, which delivers the predicted biological activity of the molecules under study.

The number of weights/number of connections defines parameter *ρ*. Parameter *ρ* holds substantial importance in determining the most suitable configuration for an ANN model [31]. It is generally advised that the value of *ρ* should fall within the range of 1 to 3. When *ρ* is less than 1, there is a risk of the network simply memorizing the training data, while values exceeding 3 may impede the networK′s ability to make accurate generalizations based on new data instances [31].

Validation of the QSAR Model: In order to evaluate the fit, stability, and predictive power of the QSAR model, we adopted various statistical parameters for modeling, internal and external validation metrics. They include R^2^ (the coefficient of determination), R^2^_adj_ (adjusted coefficient of determination), MSE (mean of squared errors of model), F_test_ (Fischer’s value), VIF (Variance Inflation Factor), and R^2^_test_ (Coefficient of determination of external test). Additionally, we assessed the internal predictive capacity of the model (Q^2^_LOO_), which uses the “Leave-One-Out” method [11], as well as the Y-randomization test parameters (R^2^_Rand_ and Q^2^_cv (Rand)_) [32,33]. For extensive validation of the predictive ability of our QSAR models, we used external validation tests that use the Tropsha and Golbraikh criteria [11,32] and rm^2^ metric analysis [33] (Table S5 of the Supplementary Materials).

### 3.4. The Applicability Domain Method

The applicability domain method was implemented in order to determine the presence of the most influential and aberrant molecules. Within this framework, any biocompound that is not within the applicability domain space of ±3 is classified as an outlier. In order to provide a comprehensive understanding of the applicability domain of the constructed QSAR models, the leverage *h*_*i*_ approach was used [33]:

(3) $$h_{i}=X_{i}{\left(X^{T}X\right)}^{-1}X_{i}^{T}$$

In this equation, *X*_*i*_ represents the training set matrix of *i*. *X* is the n × k descriptor matrix of the training set compound, and *X*^*T*^ is the transpose of the training set (*X*). Additionally, *X*^*T*^_*i*_ corresponds to the transpose matrix *X*_*i*_, which is utilized for constructing the model. It is essential to note that the warning lever, h*, serves as the threshold values used to assess the presence of influential molecules. h* is expressed as

(4) $$h^{*}=\frac{3(j+1)}{m}$$

where *j* corresponds to the total number of descriptors within the built model, while *m* represents the number of compounds that constitute the training set.

Through the use of Equations (2) and (3), the applicability domain of the constructed QSAR models is ensured. Indeed, an accurate assessment of outlier and influential molecules is performed, thereby enhancing the overall reliability and validity of the models. In addition, they allow for a more nuanced understanding of their strengths and limitations, and their applicability and reliability. Moreover, the use of the leverage *h_i_* approach further enhances the applicability domain approach by providing a quantitative measure of the influence of each descriptor on the overall model. This information proves crucial in understanding the underlying factors and variables that contribute to the model’s performance and predictions [9].

### 3.5. Molecular Docking Study

Molecular docking is a widely used computational method to predict the binding poses and energies of ligands to target proteins. However, discrepancies often occur between docking-predicted binding energies and experimentally measured affinities due to several limitations. Docking tools rely on simplified scoring functions that estimate binding energies but often overlook critical factors such as entropy, protein flexibility, and water-mediated interactions. For example, Wang et al. [34] reported significant deviations in AutoDock-predicted ΔG values due to inadequate modeling of solvation effects. The rigid receptor assumption further reduces accuracy, as proteins frequently undergo conformational changes during binding, as highlighted by Mobley and Dill [35]. Experimental variability, influenced by factors like pH and temperature, also complicates direct comparisons with docking predictions https://doi.org/10.3389/fmolb.2021.712085. These challenges highlight the necessity of conducting experimental studies to confirm and validate the theoretical findings.

**Figure 11:**
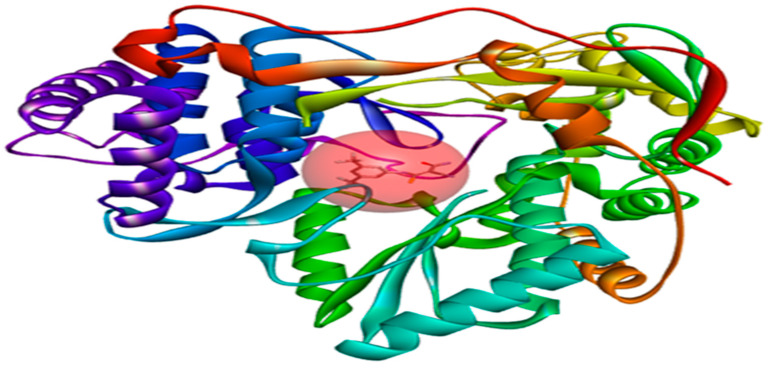
Grid box visualization of the receptor (2IJN) complexed with ligand **221**.

First, the compounds under study were optimized using the Avogadro software program 1. 1. 0 [36], employing the MMFF94 force field. In addition, the crystal structure of NS5B polymerase receptor (PDB ID: 2IJN, Figure 11), with a resolution of 2.2 Å, was optimized using the Swiss-PDB Viewer 4.1 software package [37], where all missed residues were added, followed by a preparation using Autodock4 tools [15]. This preparation involved the removal of heteroatoms and water molecules, addition of polar hydrogens, and calculation of Gasteiger charges [38]. The binding site for molecular docking was determined based on the initial position of the co-crystallized ligand [39].The coordinates of the grid box covering the active site of the receptor were set to x = 8.816Å, y = 42.819Å, and z =49.509Å, with a size of 30Å^3^ and a space center of 0.375Å (Figure 11). Subsequently, the optimized ligands and a reference compound were docked into the receptor’s binding site to unveil the interaction modes of the ligand–receptor complexes. The analysis included exploring spatial orientations for ligand attachment to the target and evaluating their binding energies [40,41].

### 3.6. ADMET Properties and Drug-likeness

An ADME analysis was conducted in order to evaluate the drug-likeness of the biocompounds that were found to have the best fit during the process of molecular docking. This was performed using SwissADME [42]. During the ADME analysis, several important characteristics were taken into consideration. They included the logarithm of partition coefficient (Log P), blood–brain barrier (BBB) permeability, the aqueous solubility (Log S), human gastrointestinal absorption (HIA), and skin permeation. In addition, the synthetic accessibility of the ligands was predicted by assigning them a numerical score ranging from 1 to 10, i.e., from relatively easy to difficult, respectively. Furthermore, various drug-likeness properties were evaluated using the following rules: Lipinski, Veber, and Ghose. Finally, the potential inhibitors were checked for toxicity using the pkCSM [43].

### 3.7. Molecular Dynamics Simulations

We performed molecular dynamics (MD) simulations to investigate the compounds identified through the ADMET analysis and molecular docking results, aiming to gain insights into their stability, flexibility, and interactions over time. Essential parameters, including root mean squared deviation (RMSD), root mean squared fluctuation (RMSF), and ligand–protein contacts, were evaluated to understand the structural integrity, reliability of binding modes, and overall behavior of the complexes [44]. The preparation of generated complexes for MD simulations included energy minimization and optimization using the OPLS3e force field, utilizing the protein preparation wizard in the Desmond package within the Schrödinger 2020–3 academic software package [45]. The simulation set up involved creating an orthorhombic system with the TIP3P water model [46], introducing Na^+^ and Cl^−^counterions to neutralize the charge, and adjusting the salt concentration to 0.15 M. Gradual heating, controlled by the Martina–Tobias–Klein method and the Nose–Hoover thermal algorithm, ensured the system reached the desired temperature below 300 K. The isothermal–isobaric ensemble (NPT) maintained the pressure at 1 bar, and the MD simulations ran for 100 ns [47].

## 4. Conclusions

We established a 2D-QSAR model focusing on the isothiazole series targeting the inhibitory mechanism on NS5B. The constructed QSAR model was employed for designing purposes and also for the identification of novel potent and selective isothiazole-based derivatives targeting NS5B inhibition. The predictions of biological activity made by the MLR and ANN models shows a strong concordance with the values obtained experimentally. The assessments conducted through LOO cross-validation technique and external validation via test set methodologies suggest that the model holds significance, robustness, and commendable predictive capacity. The outcomes of molecular docking concerning the newly formulated inhibitors elucidated the binding configurations established between the most efficient compounds (**N7**, **N8**, **N9**, and **N10**) and this protein, showcasing notably high binding affinity scores (> 8 kcal/mol in absolute values), all surpassing the reference ligand 221. This enzyme–ligand complex stability is believed to be due to multiple and various weak interactions established between these compounds and the active site of the enzyme, such as H-bond and hydrophobic interactions with the amino acid residues forming this active site. Furthermore, ADME data have illustrated that the newly designed ligands **N7**, **N8**, **N9**, and **N10** have favorable pharmacokinetic properties. Lastly, molecular dynamics simulations provided an understanding of the compounds’ behaviors interacting with this enzyme at the molecular level. Moreover, our analyses indicated the promising potential of these compounds to serve as effective inhibitors of NS5B, thereby opening up new possibilities for drug development in combating HCV.

## Figures and Tables

**Table 1:** Two-dimensional molecular structures of the studied isothiazole derivatives [5,6], along with their corresponding experimental (pIC_50_Exp), residual (pIC_50_Res) and predicted (pIC_50_Pred) activities using (MLR) analysis. * corresponds to test set compounds.

No	Structure	pIC_50_ Exp.	pIC_50_ Pred MLR	pIC_50_ Res. MLR	No	Structure	pIC_50_ Exp.	pIC_50_ Pred MLR	pIC_50_ Res. MLR	No	Structure	pIC_50_ Exp.	pIC_50_ Pred MLR	pIC_50_ Res. MLR
1 *	** 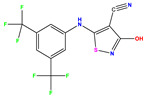 **	5.229	6.055	−0.826	**14**	** 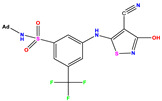 **	5.456	5.913	−0.457	**27**	** 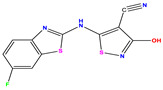 **	4.357	5.903	−1.546
2 *	** 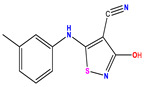 **	4	4.256	−0.256	**15**	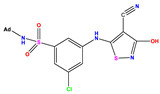	5.745	5.920	−0.175	28 *	** 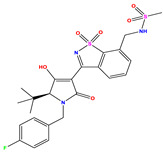 **	8.523	8.251	0.273
**3**	** 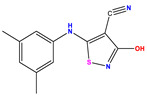 **	4	4.248	−0.248	**16**	** 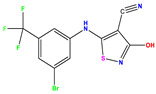 **	6.523	6.423	0.100	**29**	** 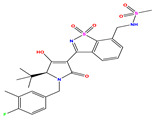 **	8.398	8.104	0.294
**4**	** 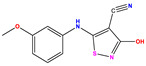 **	4	4.536	−0.536	17 *	** 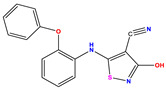 **	5.886	5.081	0.805	**30**	** 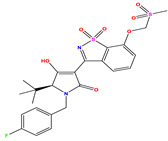 **	8.046	7.406	0.640
**5**	** 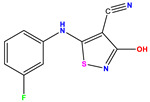 **	6.046	5.162	0.884	**18**	** 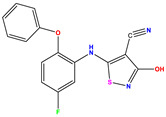 **	6.301	5.963	0.338	**31**	** 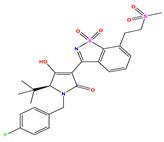 **	8.398	8.468	−0.070
**6**	** 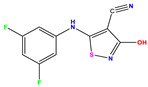 **	6.523	6.012	0.511	19 *	** 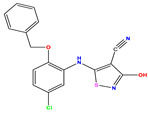 **	6.000	5.428	0.572	32 *	** 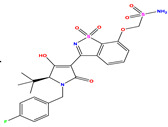 **	8.222	8.194	0.028
**7**	** 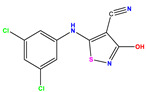 **	6.699	6.078	0.621	**20**	** 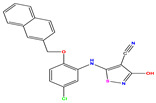 **	5.194	6.022	−0.828	**33**	** 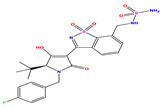 **	8.523	7.970	0.554
**8**	** 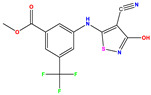 **	5.699	5.625	0.074	**21**	** 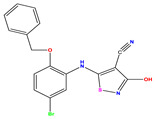 **	6.523	5.793	0.731	**34**	** 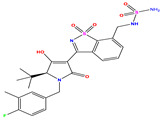 **	8.523	7.813	0.711
**9**	** 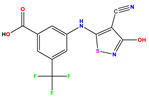 **	5.824	5.579	0.245	**22**	** 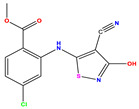 **	5.328	5.672	−0.344	35 *	** 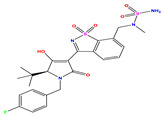 **	9.000	7.980	1.020
**10**	** 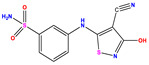 **	5.013	5.187	−0.174	**23**	** 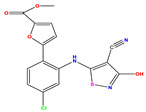 **	5.7450	6.479	−0.734	**36**	** 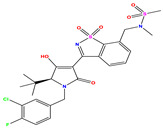 **	8.000	8.199	−0.199
**11**	** 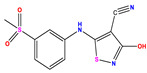 **	5.013	5.654	−0.641	**24**	** 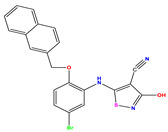 **	5.770	6.329	−0.559	**37**	** 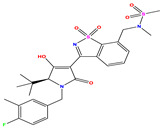 **	8.301	8.774	−0.473
**12**	** 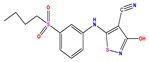 **	5.081	5.234	−0.153	25 *	** 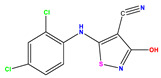 **	6.155	6.000	0.155	**38**	** 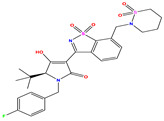 **	7.921	8.252	−0.331
**13**	** 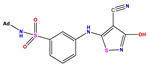 **	5.854	5.039	0.815	**26**	** 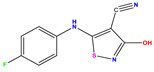 **	5.921	4.974	0.947					

**Table 2:** Molecular descriptor values employed in the regression analysis.

Compound	ET × 10^3^ (a.u.)	D	Log D	E_HOMO_ (Hartree)	E_LUMO_ (Hartree)	μ (Debye)	MW (amu)	Log P	H (kcal/mol)	SAG (Å^2^)	MV (Å^3^)	MR (Å^3^)	Pol (Å^3^)	PSA	HBA	HBD	NRB
**1**	−46.177	1.67	2.82	−0.242	−0.071	1.810	353.24	2.62	−14.53	477.12	771.34	73.92	25.87	68.94	4	2	4
**2**	−28.905	1.41	1.58	−0.219	−0.048	4.288	231.27	1.63	−14.70	419.16	666.43	67.77	24.58	68.94	4	2	2
**3**	−29.975	1.36	2.09	−0.217	−0.047	4.723	245.30	1.78	−13.50	444.25	718.33	72.05	26.42	68.94	4	2	2
**4**	−30.951	1.45	0.91	−0.218	−0.048	3.136	247.27	0.48	−17.46	438.57	691.79	69.86	25.22	78.17	5	2	3
**5**	−30.535	1.54	1.21	−0.227	−0.056	2.783	235.24	0.88	−15.60	396.96	624.19	63.61	22.66	68.94	4	2	2
**6**	−33.235	1.60	1.35	−0.234	−0.061	2.473	253.23	0.28	−15.27	402.73	633.09	63.74	22.56	68.94	4	2	2
**7**	−52.847	1.68	2.28	−0.237	−0.065	2.169	286.14	1.03	−15.13	438.73	699.99	72.92	26.60	68.94	4	2	2
**8**	−43.207	1.59	1.95	−0.237	−0.077	1.583	343.28	1.46	−15.59	517.44	831.23	79.47	28.70	95.24	6	2	5
**9**	−42.137	1.73	1.11	−0.238	−0.079	0.160	329.25	1.43	−20.12	477.16	768.35	74.70	26.87	106.20	6	3	4
**10**	−44.268	1.73	0.09	−0.237	−0.067	6.065	296.32	−0.38	−22.14	466.46	750.30	76.74	25.24	129.10	7	4	3
**11**	−43.832	1.61	0.09	−0.236	−0.066	5.411	295.33	−0.12	−18.31	473.90	763.51	77.15	25.72	103.10	6	2	3
**12**	−47.042	1.45	1.38	−0.235	−0.065	5.266	337.41	1.09	−16.55	566.21	922.15	91.03	31.22	103.10	6	2	6
**13**	−54.868	1.52	1.99	−0.230	−0.059	4.570	430.54	1.56	−17.18	631.84	1102.08	116.07	41.26	115.10	7	3	4
**14**	−64.039	1.59	2.87	−0.241	−0.069	5.493	498.54	2.12	−16.33	635.45	1140.42	121.28	42.83	115.10	7	3	5
**15**	−67.374	1.58	2.59	−0.238	−0.066	3.742	464.98	1.33	−17.08	639.26	1124.87	120.78	43.19	115.10	7	3	4
**16**	−106.968	1.89	2.71	−0.239	−0.069	2.538	364.14	2.10	−14.74	467.63	754.57	76.24	26.94	68.94	4	2	3
**17**	−36.168	1.45	2.57	−0.210	−0.052	3.185	309.34	0.98	−17.73	494.08	835.14	94.45	33.04	78.17	5	2	4
**18**	−38.868	1.50	2.71	−0.212	−0.057	2.209	327.33	0.37	−17.48	500.90	845.54	94.58	32.95	78.17	5	2	4
**19**	−49.745	1.48	3.34	−0.217	−0.052	4.245	357.81	1.28	−17.61	538.48	926.12	103.33	36.81	78.17	5	2	5
**20**	−53.926	1.49	4.23	−0.222	−0.053	4.547	407.87	1.35	−18.02	605.89	1050.80	121.53	42.99	78.17	5	2	5
**21**	−107.201	1.66	3.40	−0.218	−0.053	3.099	402.27	1.55	−17.62	553.40	947.86	106.15	37.51	78.17	5	2	5
**22**	−46.542	1.59	2.98	−0.232	−0.075	1.317	309.73	0.67	−14.85	458.40	764.45	78.97	29.07	95.24	6	2	4
**23**	−52.769	1.59	2.30	−0.225	−0.075	2.307	375.79	−0.36	−17.73	569.35	948.94	99.61	35.89	108.40	7	2	5
**24**	−111.382	1.64	4.39	−0.221	−0.053	4.531	452.32	1.62	−17.96	613.82	1069.24	124.34	43.69	78.17	5	2	5
**25**	−52.847	1.68	2.28	−0.231	−0.063	3.524	286.14	1.03	−16.00	430.48	690.94	72.92	26.60	68.94	4	2	2
**26**	−30.535	1.54	1.21	−0.223	−0.052	2.814	235.24	0.88	−15.67	397.98	624.03	63.61	22.66	68.94	4	2	2
**27**	−43.881	1.72	2.02	−0.226	−0.073	3.893	292.31	0.91	−16.20	454.15	720.65	76.94	27.65	81.83	5	2	2
**28**	−66.447	1.46	1.85	−0.249	−0.095	14.364	535.61	0.07	−12.75	732.13	1312.48	140.68	46.92	133.20	8	2	6
**29**	−67.517	1.44	1.33	−0.239	−0.091	15.026	549.63	0.23	−11.23	767.26	1362.44	144.97	48.75	133.20	8	2	6
**30**	−66.988	1.46	1.81	−0.249	−0.089	8.792	536.59	−0.10	−12.24	743.98	1308.79	138.80	46.20	130.40	9	1	7
**31**	−66.012	1.42	1.39	−0.247	−0.105	7.393	534.62	0.45	−9.20	744.36	1322.28	141.38	47.40	121.20	8	1	7
**32**	−67.424	1.54	2.05	−0.248	−0.102	16.776	537.58	−0.36	−15.97	719.88	1289.88	138.39	45.72	156.40	9	2	7
**33**	−66.884	1.55	2.30	−0.246	−0.100	15.592	536.59	−0.19	−14.50	726.61	1290.06	140.27	46.43	159.20	9	3	6
**34**	−67.954	1.52	1.78	−0.239	−0.095	13.200	550.62	−0.03	−14.36	748.20	1339.66	144.55	48.27	159.20	9	3	6
**35**	−67.953	1.49	2.07	−0.244	−0.095	13.237	550.02	−0.27	−12.13	735.69	1327.01	145.17	47.27	150.40	9	2	6
**36**	−80.023	1.47	1.03	−0.248	−0.092	10.387	584.08	−0.24	−9.47	760.07	1380.74	150.30	50.68	124.40	9	1	6
**37**	−67.518	1.40	1.10	−0.248	−0.100	3.504	563.66	0.14	−7.42	772.22	1390.55	149.86	50.59	124.40	8	1	6
**38**	−69.624	1.47	1.06	−0.244	−0.089	11.480	575.67	0.31	−10.15	778.96	1413.42	153.12	51.65	124.40	8	1	6

**Table 3:** Variance Inflation Factor (VIF) of molecular descriptors and the Pearson correlation.

(Variables)	MW (amu)	Log P	HBA	E_LUMO_ (Hartree_)_	VIF
MW	1				5.197
Log P	−0.239	1			2.060
HBA	0.850	−0.548	1		7.402
E_LUMO_	−0.748	0.516	−0.832	1	3.506

**Table 4:** MLR parameters of model.

Average R	0.351
Average R^2^	0.139
Average Q^2^	−0.243
cRp^2^	0.747

**Table 5:** The matrix r^2^m and the criterion of Golbraikh and Tropsha.

Parameter	Threshold Score	Model Score
Q^2^_LOO_	Q^2^_Loo_ > 0.5	0.737
$R_{pred}^{2}$	$R_{pred}^{2}>0.6$	0.874
$\frac{\left(R^{2}-R_{0}^{2}\right)}{R^{2}}$	$\frac{\left(R^{2}-R_{0}^{2}\right)}{R^{2}}$ < 0.1	0.011
$\frac{\left(R^{2}-{R^{\prime }}_{0}^{2}\right)}{R^{2}}$	$\frac{\left(R^{2}-{R^{\prime }}_{0}^{2}\right)}{R^{2}}$ < 0.1	0.064
K	0.85 ≤ K ≤ 1.15	1.037
K′	0.85 ≤ K′ ≤ 1.15	0.958
$\left|R_{0}^{2}-{R^{\prime }}_{0}^{2}\right|$	$\left|R_{0}^{2}-{R^{\prime }}_{0}^{2}\right|$ < 0.3	0.198
r^2^_m_	r^2^_m_ > 0.5	0.766
r’^2^_m_	r’^2^_m_> 0.5	0.623

**Table 6:** Predicted pIC_50_s estimated using ANN.

Compounds	Pred.pIC_50_ ANN	Compounds	Pred.pIC_50_ ANN
**M1**	6.30	**M20**	5.47
**M2**	3.90	**M21**	6.03
**M3**	4.09	**M22**	5.20
**M4**	4.08	**M23**	5.78
**M5**	5.10	**M24**	5.93
**M6**	6.58	**M25**	6.69
**M7**	6.56	**M26**	5.86
**M8**	5.70	**M27**	4.59
**M9**	5.83	**M28**	8.22
**M10**	5.03	**M29**	8.25
**M11**	4.97	**M30**	8.08
**M12**	5.09	**M31**	8.41
**M13**	5.85	**M32**	8.39
**M14**	5.48	**M33**	8.52
**M15**	5.74	**M34**	8.45
**M16**	6.54	**M35**	8.38
**M17**	4.71	**M36**	8.03
**M18**	6.24	**M37**	8.37
**M19**	5.34	**M38**	7.97

**Table 7:** Two-dimensional structure, calculated descriptor data, predicted activity, and leverage effects noted (h) associated with newly formulated compounds.

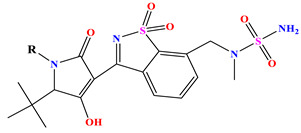							
**Compound**	**R Substituent**	**MW**	**Log P**	**HBA**	**E_LUMO_** **(Hartree)**	**pIC_50_**	**h**
**N7**	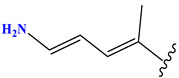	523.62	−0.73	8	−0.101	8.968	0.281
**N8**	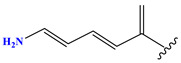	535.63	−0.33	8	−0.098	8.657	0.191
**N9**	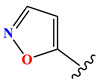	509.55	0.64	8	−0.105	8.061	0.133
**N10**	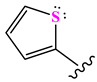	524.62	0.90	7	−0.092	8.097	0.144

**Table 8:** Binding scores and visualizations for ligand–receptor complexes.

Compounds	Score (kcal/Mol)	3D Visualization	2D Visualization
**N7-2IJN**	**−8**	** 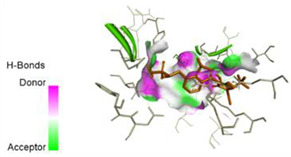 **	** 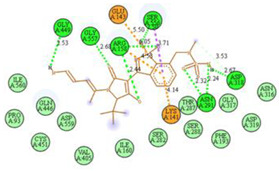 **
**N8-2IJN**	**−8.1**	** 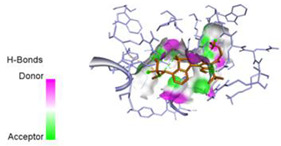 **	** 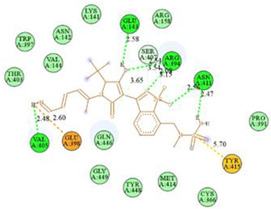 **
**N9-2IJN**	**−9.3**	** 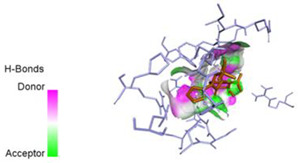 **	** 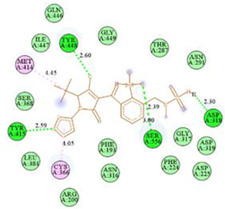 **
**N10-2IJN**	**−8.5**	** 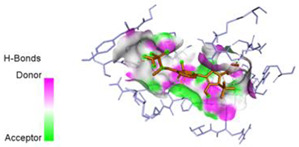 **	** 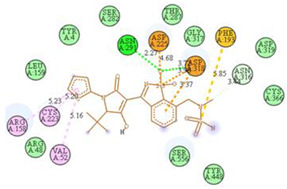 **
**Reference** **(221)-2IJN**	**−7.5**	** 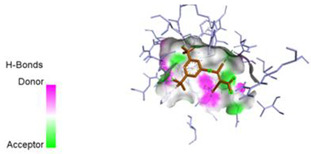 **	** 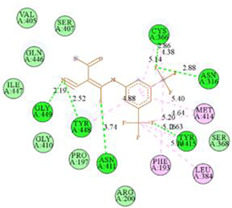 **
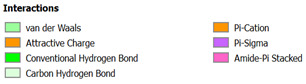			

**Table 9:** Drug-likeness and ADMET prediction of the newly inhibitors N7, N8, N9, N10 and the co-crystalized compound **221**.

Parameters	N7	N8	N9	N10	Compound 221
MW	523.63	535.64	509.56	524.63	357.28
Rotatable Bonds	6	8	6	6	6
H-bond acceptors	7	7	8	7	4
H-bond donors	3	3	2	2	3
TPSA	193.22	193.22	193.23	195.44	117.71
Log P	1.2585	4.36	1.0749	2.1484	3.79
BBB permeant	−1.137	−1.148	−1.861	−1.626	−1.183
P-gp substrate	Yes	Yes	No	No	No
LogK_p_	−2.794	−2.786	−2.752	−2.743	−2.818
CYP1A2 inhibitor	No	No	No	No	Yes
CYP2C19 inhibitor	No	No	No	No	Yes
CYP2C9 inhibitor	No	No	No	No	No
CYP2D6 inhibitor	No	No	No	No	No
CYP3A4 inhibitor	Yes	Yes	No	Yes	No
AMES toxicity	No	No	Yes	No	No
hERG I inhibitor	No	No	No	No	No
Skin sensitization	No	No	No	No	No

## Data Availability

The original contributions presented in the study are included in the article/Supplementary Material, further inquiries can be directed to the corresponding author/s.

## Supplementary Materials

The following supporting information can be downloaded at: https://www.mdpi.com/article/10.3390/ph17121712/s1, Table S1: Y-randomization test results; Table S2: Smiles of the newly formulated compounds (N7, N8, N9, N10, and 221); Table S3: Overview of interactions between docked ligands and NS5B Polymerase (PDB ID: 2IJN). The table presents the participating residues, interaction types, and distances (in angstroms, Å) for each complex; Table S4: The optimized structures of the studied isothiazole derivatives; Table S5: The five criteria employed to assess the predictive capability of the model as outlined by Tropsha and Golbraikh pertain to the external validation set.

## References

[1] Adedotun I.O., Abdul-Hammed M., Hamzat B.A., Adepoju A.J., Akinboade M.W., Afolabi T.I., Ismail U.T. (2022). Molecular docking, ADMET analysis, and bioactivity studies of phytochemicals from *Phyllanthus niruri* as potential inhibitors of hepatitis C virus NSB5 polymerase. J. Indian Chem. Soc..

[2] Calland N., Dubuisson J., Rouillé Y., Séron K. (2012). Hepatitis C virus and natural compounds: A new antiviral approach?. Viruses.

[3] Aloui M., Er-rajy M., Imtara H., Goudzal A., Zarougui S., El fadili M., Arthur D.E., Mothana R.A., Noman O.M., Tarayrah M. (2024). QSAR modelling, molecular docking, molecular dynamic and ADMET prediction of pyrrolopyrimidine derivatives as novel Bruton’s tyrosine kinase (BTK) inhibitors. Saudi Pharm. J..

[4] Ejeh S., Uzairu A., Shallangwa G.A., Abechi S.E., Ibrahim M.T., Ramu R. (2023). Cheminformatics study of some indole compounds through QSAR modeling, ADME prediction, molecular docking, and molecular dynamic simulation to identify novel inhibitors of HCV NS5B proteas. J. Indian Chem. Soc..

[5] Yan S., Appleby T., Gunic E., Shim J.H., Tasu T., Kim H., Rong F., Chen H., Hamatake R., Wu J.Z. (2007). Isothiazoles as active-site inhibitors of HCV NS5B polymerase. Bioorganic Med. Chem. Lett..

[6] de Vicente J., Hendricks R.T., Smith D.B., Fell J.B., Fischer J., Spencer S.R., Stengel P.J., Mohr P., Robinson J.E., Blake J.F. (2009). Non-nucleoside inhibitors of HCV polymerase NS5B. Part 4: Structure-based design, synthesis, and biological evaluation of benzo[d]isothiazole-1,1-dioxides. Bioorganic Med. Chem. Lett..

[7] Singh R., Kumar P., Sindhu J., Devi M., Kumar A., Lal S., Singh D. (2023). Parsing structural fragments of thiazolidin-4-one based α-amylase inhibitors: A combined approach employing in vitro colorimetric screening and GA-MLR based QSAR modelling supported by molecular docking, molecular dynamics simulation and ADMET studies. Comput. Biol. Med..

[8] Alloui M., Belaidi S., Othmani H., Jaidane N.E., Hochlaf M. (2018). Imidazole derivatives as angiotensin II AT1 receptor blockers: Benchmarks, drug-like calculations and quantitative structure-activity relationships modeling. Chem. Phys. Lett..

[9] Khamouli S., Belaidi S., Bakhouch M., Chtita S., Hashmi M.A., Qais F.A. (2022). QSAR modeling, molecular docking, ADMET prediction and molecular dynamics simulations of some 6-arylquinazolin-4-amine derivatives as DYRK1A inhibitors. J. Mol. Struct..

[10] Dahmani R., Manachou M., Belaidi S., Chtita S., Boughdiri S. (2021). Structural characterization and QSAR modeling of 1, 2, 4-triazole derivatives as α-glucosidase inhibitors. New J. Chem..

[11] Golbraikh A., Tropsha A. (2002). Beware of q^2^!. J. Mol. Graph. Model..

[12] Dermeche K., Tchouar N., S Belaidi S., Salah T. (2015). Qualitative Structure-Activity Relationships and 2D-QSAR Modeling of TNF-α Inhibition by Thalidomide Derivatives. J. Bionanoscience.

[13] Almi I., Belaidi S., Zerroug E., Alloui M., Said R.B., Linguerri R., Hochlaf M. (2020). QSAR investigations and structure-based virtual screening on a series of nitrobenzoxadiazole derivatives targeting human glutathione-S- transferases. J. Mol. Struct..

[14] Soualmia F., Belaidi S., Tchouar N., Lanez T., Boudergua S. (2021). QSAR Studies and Structure Property/Activity Relationships Applied in Pyrazine Derivatives as Antiproliferative Agents Against the BGC823. Acta Chim. Slov..

[15] Morris G.M., Huey R., Lindstrom W., Sanner M.F., Belew R.K., Goodsell D.S., Olson A.J. (2009). AutoDock4 and AutoDockTools4: Automated docking with selective receptor flexibility. J. Comput. Chem..

[16] Fattouche M., Belaidi S., Ouassaf M., Chtita S., Al-Mogren M.M., Hochlaf M. (2024). Computational studies of pyrimidine derivatives as inhibitors of human σ1 receptor using 3D-QSAR analysis, molecular docking, ADMET properties and DFT investigation. Chem. Phys. Impact..

[17] Lipinski C.A., Lombardo F., Dominy B.W., Feeney P.J. (2001). Experimental and computational approaches to estimate solubility and permeability in drug discovery and development settings. Adv. Drug Deliv. Rev..

[18] Ghose A.K., Viswanadhan V.N., Wendoloski J.J. (1999). A Knowledge-Based Approach in Designing Combinatorial or Medicinal Chemistry Libraries for Drug Discovery. 1. A Qualitative and Quantitative Characterization of Known Drug Databases. J. Comb. Chem..

[19] Veber D.F., Johnson S.R., Cheng H.Y., Smith B.R., Ward K.W., Kapple K.D. (2002). Molecular Properties That Influence the Oral Bioavailability of Drug Candidates. J. Med. Chem..

[20] Mishra S., Dahima R. (2019). In vitro ADME studies of TUG-891, a GPR-120 inhibitor using Swiss ADME predictor. J. Drug Deliv. Ther..

[21] Brunton L., Chabner B.A., Knollman B. (2011). Goodman and Gilman’s The Pharmacological Basis of Therapeutics.

[22] Nour H., Abdou A., Belaidi S., Jamal J.E., Elmakssoudi A., Dakir M., Chtita S. (2022). Discovery of promising cholinesterase inhibitors for Alzheimer’s disease treatment through DFT, docking, and molecular dynamics studies of eugenol derivatives. J. Chin. Chem. Soc..

[23] Chem Office. https://support.revvitysignals.com/hc/en-us/articles/4408268473492.

[24] Belaidi S., Salah T., Melkemi N., Sinha L., Prasad O. (2015). Structure Activity Relationship and Quantitative Structure-Activity Relationships Modeling of Antitrypanosomal Activities of Alkyldiamine Cryptolepine Derivatives. J. Comput. Theor. Nanosci..

[25] (2008). HyperChem (Molecular Modelling System).

[26] Frisch M.J., Trucks G.W., Schlegel H.B., Scuseria G.E., Robb M.A., Cheeseman J.R., Scalmani G., Barone V., Mennucci B., Petersson G.A. (2010). Gaussian09, revision B.01..

[27] Chemaxon. https://dl.chemaxon.com/docs/HTML/docs164110/MarvinSketch.html.

[28] Apablaza G., Montoya L., Morales-Verdejo C., Mellado M., Cuellar M., Lagos C.F., Soto-Delgado J., Chung H., Pessoa-Mahana C.D., Mella J. (2017). 2D-QSAR and 3D-QSAR/CoMSIA Studies on a Series of (R)-2-((2-(1H-Indol-2-yl)ethyl)amino)-1-Phenylethan-1-ol with Human β_3_-Adrenergic Activity. Molecules.

[29] *XLSTAT*, version 2014.5.03; Addinsoft: Paris, France.

[30] Chtita S., Belhassan A., Bakhouch M., Taourati A.I., Aouidate A., Belaidi S., Moutaabbid M., Belaaouad S., Bouachrine M., Lakhlifi T. (2021). QSAR study of unsymmetrical aromatic disulfides as potent avian SARS-CoV main protease inhibitors using quantum chemical descriptors and statistical methods. Chemom. Intell. Lab. Syst..

[31] Aggoun S., Belaidi S., Ghamri M., Abchir O., Chtita S. (2024). Artificial Neural Network and Multiple Regression Analysis Applied to 2D-QSAR Studies: The Case of Imidazolidine-2,4-dione as PTP1B Inhibiton. Philipp. J. Sci..

[32] Tropsha A. (2010). Best practices for QSAR model development, validation, and exploitation. Mol. Inform..

[33] Roy K., Chakraborty P., Mitra I., Ojha P.K., Kar S., Das R.N. (2013). Some Case Studies on Application of “rm2” Metrics for Judging Quality of Quantitative Structure-Activity Relationship Predictions: Emphasis on Scaling of Response Data. J. Comp. Chem..

[34] Wang Z., Sun H., Yao X., Li D., Xu L., Li Y., Tian S., Hou T. (2016). Comprehensive evaluation of ten docking programs on a diverse set of protein-ligand complexes: The prediction accuracy of sampling power and scoring power. Phys. Chem. Chem. Phys..

[35] Mobley D.L., Graves A.P., Chodera J.D., McReynolds A.C., Shoichet B.K., Dill K.A. (2007). Predicting absolute ligand binding free energies to a simple model site. J. Mol. Biol..

[36] Hanwell M.D., Curtis D.E., Lonie D.C., Vandermeersch T., Zurek E., Hutchison G.R. (2012). Avogadro: An advanced semantic chemical editor, visualization, and analysis platform. J. Chem. Inform..

[37] Guex N., Peitsch M.C. (1997). SWISS-MODEL and the Swiss-PdbViewer: An environment for comparative protein modeling. J. Electrophor..

[38] Khamouli S., Belaidi S., Ouassaf M., Lanez T., Belaaouad S., Chtita S. (2022). Multi-combined 3D-QSAR, docking molecular and ADMET prediction of 5-azaindazole derivatives as LRRK2 tyrosine kinase inhibitors. J. Biomol. Struct. Dyn..

[39] Abchir O., Daoui O., Belaidi S., Ouassaf M., Qais F.A., ElKhattabi S., Belaaouad S., Chtita S. (2022). Design of novel benzimidazole derivatives as potential α-amylase inhibitors using QSAR, pharmacokinetics, molecular docking, and Molecular Dynamics Simulation Studies. J. Mol. Model..

[40] Nour H., Abchir O., Belaidi S., Chtita S. (2022). Research of new acetylcholinesterase inhibitors based on QSAR and molecular docking studies of benzene-based carbamate derivatives. J. Struct. Chem..

[41] Abchir O., Nour H., Daoui O., Yamari I., ElKhattabi S., El Kouali M., Talbi M., Errougui A., Chtita S. (2023). Structure-based virtual screening, ADMET analysis, and molecular dynamics simulation of Moroccan natural compounds as candidates for the SARS-COV-2 inhibitors. J. Nat. Prod. Res..

[42] Daina A., Michielin O., Zoete V. (2017). SwissADME: A free web tool to evaluate pharmacokinetics, drug-likeness and medicinal chemistry friendliness of small molecules. Sci. Rep..

[43] Pkcsm. 25 July 2022. https://biosig.lab.uq.edu.au/pkcsm/prediction.

[44] Derki N.E.H., Kerassa A., Belaidi S., Derki M., Yamari I., Samadi A., Chtita S. (2024). Computer-Aided Strategy on 5-(Substituted benzylidene) Thiazolidine-2, 4-Diones to Develop New and Potent PTP1B Inhibitors: QSAR Modeling, Molecular Docking, Molecular Dynamics. Molecules.

[45] (2017). Schrodinger, System, Maestro-Desmond Interoperability Tools, Software (2021).

[46] Mouhsin M., Abchir O., El Otmani F.S., Oumghar A.A., Oubenali M., Chtita S., Mbarki M., Gamouh A. (2023). Identification of novel NLRP3 inhibitors: A comprehensive approach using 2D-QSAR, molecular docking, molecular dynamics simulation and drug-likeness evaluation. Chem. Pap..

[47] Mohamed A.S., Elmi A., Spina R., Kordofani M.A., Laurain-Mattar D., Nour H., Abchir O., Chtita S. (2023). In vitro and in silico analysis for elucidation of antioxidant potential of Djiboutian Avicennia marina (Forsk.) Vierh. Phytochemicals. J. Biomol. Struct. Dyn..

