# Supplementary material for: ANN-QSAR, Molecular Docking, ADMET Predictions, and Molecular Dynamics Studies of Isothiazole Derivatives to Design New and Selective Inhibitors of HCV Polymerase NS5B
Source: Pharmaceuticals (Basel). 2024 Dec 18;17(12):1712. doi: 10.3390/ph17121712 (PMC11678770; doi:10.3390/ph17121712)
Supplement: Supplementary file 1 [file pharmaceuticals-17-01712-s001.zip › pharmaceuticals-3334533-supplementary.pdf]

Supplementary Materials

Table S1. Y-randomization test results.

Model	R	R^2	Q^2(LOO)	Model	R	R^2	Q^2(LOO)
Original	0.900477	0.810859	0.736805	Random 51	0.504745	0.254767	-0.08221
Random 1	0.243788	0.059432	-0.39108	Random 52	0.553631	0.306508	0.004439
Random 2	0.322929	0.104283	-0.3206	Random 53	0.46072	0.212263	-0.13188
Random 3	0.295088	0.087077	-0.40218	Random 54	0.260774	0.068003	-0.31452
Random 4	0.217354	0.047243	-0.3149	Random 55	0.41301	0.170577	-0.19141
Random 5	0.175573	0.030826	-0.37556	Random 56	0.487534	0.237689	-0.10276
Random 6	0.27374	0.074934	-0.30647	Random 57	0.248998	0.062	-0.35919
Random 7	0.160077	0.025625	-0.40923	Random 58	0.316656	0.100271	-0.27017
Random 8	0.450373	0.202836	-0.15087	Random 59	0.19816	0.039267	-0.36096
Random 9	0.187875	0.035297	-0.40176	Random 60	0.285044	0.08125	-0.3715
Random 10	0.34207	0.117012	-0.31844	Random 61	0.550183	0.302701	0.028133
Random 11	0.248114	0.061561	-0.33224	Random 62	0.222449	0.049483	-0.32945
Random 12	0.390673	0.152626	-0.21959	Random 63	0.361571	0.130733	-0.2231
Random 13	0.360185	0.129733	-0.30334	Random 64	0.311726	0.097173	-0.32241
Random 14	0.232172	0.053904	-0.37395	Random 65	0.26617	0.070846	-0.36257
Random 15	0.346214	0.119864	-0.30628	Random 66	0.453018	0.205226	-0.16409
Random 16	0.348056	0.121143	-0.26079	Random 67	0.510382	0.26049	-0.08114
Random 17	0.205298	0.042147	-0.39689	Random 68	0.295253	0.087174	-0.3491
Random 18	0.324988	0.105617	-0.33439	Random 69	0.409611	0.167781	-0.16974
Random 19	0.357948	0.128127	-0.25472	Random 70	0.29897	0.089383	-0.27924
Random 20	0.300414	0.090248	-0.32115	Random 71	0.30422	0.09255	-0.37002
Random 21	0.436394	0.19044	-0.14224	Random 72	0.493621	0.243661	-0.10136
Random 22	0.309684	0.095904	-0.41989	Random 73	0.135943	0.018481	-0.34053
Random 23	0.225482	0.050842	-0.4692	Random 74	0.278853	0.077759	-0.26984
Random 24	0.309854	0.096009	-0.36042	Random 75	0.625186	0.390857	0.073371
Random 25	0.473606	0.224303	-0.11098	Random 76	0.739151	0.546344	0.362071
Random 26	0.294992	0.08702	-0.38416	Random 77	0.22513	0.050683	-0.36564
Random 27	0.471936	0.222723	-0.11792	Random 78	0.296597	0.08797	-0.29569
Random 28	0.288401	0.083175	-0.329	Random 79	0.618847	0.382971	0.163572
Random 29	0.140741	0.019808	-0.49752	Random 80	0.158578	0.025147	-0.46306
Random 30	0.256251	0.065665	-0.29382	Random 81	0.5234	0.273947	-0.00026
Random 31	0.484702	0.234936	-0.063	Random 82	0.268249	0.071958	-0.36624
Random 32	0.427558	0.182806	-0.18509	Random 83	0.488543	0.238674	-0.05018
Random 33	0.281576	0.079285	-0.37913	Random 84	0.177427	0.03148	-0.46468
Random 34	0.239749	0.05748	-0.30041	Random 85	0.288317	0.083126	-0.30679
Random 35	0.494576	0.244605	-0.05825	Random 86	0.249473	0.062237	-0.34913
Random 36	0.351027	0.12322	-0.24115	Random 87	0.351863	0.123807	-0.20897
Random 37	0.314703	0.099038	-0.33336	Random 88	0.422794	0.178755	-0.16309
Random 38	0.214723	0.046106	-0.34198	Random 89	0.622547	0.387564	0.063454

Random 39	0.532772	0.283846	-0.00031	Random 90	0.470244	0.221129	-0.14671
Random 40	0.636069	0.404584	0.150731	Random 91	0.261264	0.068259	-0.31841
Random 41	0.397367	0.157901	-0.21317	Random 92	0.423453	0.179313	-0.21767
Random 42	0.392794	0.154287	-0.26132	Random 93	0.220134	0.048459	-0.31224
Random 43	0.327213	0.107069	-0.28016	Random 94	0.346026	0.119734	-0.21094
Random 44	0.359655	0.129352	-0.23841	Random 95	0.363526	0.132151	-0.29301
Random 45	0.373003	0.139132	-0.29172	Random 96	0.601061	0.361274	0.105196
Random 46	0.531958	0.282979	-0.02344	Random 97	0.225333	0.050775	-0.42204
Random 47	0.454153	0.206255	-0.18862	Random 98	0.324309	0.105176	-0.27744
Random 48	0.554262	0.307206	0.023232	Random 99	0.274549	0.075377	-0.35476
Random 49	0.466175	0.217319	-0.13514	Random 100	0.521494	0.271956	-0.05447
Random 50	0.454335	0.20642	-0.13512	Random 101	0.239209	0.057221	-0.39579

Table S2. Smiles of the newly formulated compounds (N7, N8, N9, N10, and 221).

	**Smile**
**N7**	[H]OC1=C(C(=O)N(\C(=C(\[H])/C(/[H])=C(\[H])N([H])[H])C([H])([H])[H])[C@@]1([H])C(C([H])([H])[H])(C([H])([H])[H])C([H])([H])[H])C1=NS(=O)(=O)c2c1c([H])c([H])c([H])c2C([H])([H])N(C([H])([H])[H])S(=O)(=O)N([H])[H]
**N8**	[H]OC1=C(C(=O)N(C(=C([H])[H])C(\[H])=C(\[H])/C(/[H])=C(/[H])N([H])[H])[C@]1([H])C(C([H])([H])[H])(C([H])([H])[H])C([H])([H])[H])C1=NS(=O)(=O)c2c1c([H])c([H])c([H])c2C([H])([H])N(C([H])([H])[H])S(=O)(=O)N([H])[H]
**N9**	[H]OC1=C(C(=O)N(C2=C([H])C([H])=NO2)[C@@]1([H])C(C([H])([H])[H])(C([H])([H])[H])C([H])([H])[H])C1=NS(=O)(=O)c2c1c([H])c([H])c([H])c2C([H])([H])N(C([H])([H])[H])S(=O)(=O)N([H])[H]
**N10**	[H]OC1=C(C(=O)N(C2=C([H])C([H])=C([H])S2)[C@@]1([H])C(C([H])([H])[H])(C([H])([H])[H])C([H])([H])[H])C1=NS(=O)(=O)c2c1c([H])c([H])c([H])c2C([H])([H])N(C([H])([H])[H])S(=O)(=O)N([H])[H]
**221**	[H]S[C@@]([H])(N([H])C1=C([H])C(=C([H])C(=C1[H])C(F)(F)F)C(F)(F)F)[C@@]([H])(C#N)C(=O)N([H])[H]

Table S3. Overview of interactions between docked ligands and NS5B Polymerase (PDB ID: 2IJN). The table presents the participating residues, interaction types, and distances (in angstroms, Å) for each complex.

Complex	Residues	Distance (Å)	Interaction Type	
**7-2IJN**	GLU143	5.49524	Electrostatic	Attractive Charge
	ARG158	2.51684	Hydrogen Bond	Conventional Hydrogen Bond
	ARG158	2.02537	Hydrogen Bond	Conventional Hydrogen Bond
	ARG158	2.43515	Hydrogen Bond	Conventional Hydrogen Bond
	ASN291	2.31834	Hydrogen Bond	Conventional Hydrogen Bond
	ASN291	2.24446	Hydrogen Bond	Conventional Hydrogen Bond
	SER556	2.49759	Hydrogen Bond	Conventional Hydrogen Bond
	GLY557	2.6766	Hydrogen Bond	Conventional Hydrogen Bond
	GLY449	2.53009	Hydrogen Bond	Conventional Hydrogen Bond
	ASP318	2.6712	Hydrogen Bond	Conventional Hydrogen Bond
	ASP318	3.52535	Hydrogen Bond	Carbon Hydrogen Bond
	LYS141	4.14492	Electrostatic	Pi-Cation
	ARG158	4.52654	Electrostatic	Pi-Cation
	SER55	3.71196	Hydrophobic	Pi-Sigma
	SER55	4.25751	Hydrophobic	Amide-Pi Stacked
**8-2IJN**	GLU398	2.60354	Hydrogen Bond; Electrostatic	Salt Bridge; Attractive Charge
	ARG394	2.54266	Hydrogen Bond	Conventional Hydrogen Bond
	ARG394	2.07506	Hydrogen Bond	Conventional Hydrogen Bond
	ASN411	2.56054	Hydrogen Bond	Conventional Hydrogen Bond
	VAL405	2.47865	Hydrogen Bond	Conventional Hydrogen Bond
	GLU143	2.58175	Hydrogen Bond	Conventional Hydrogen Bond
	SER407	3.64908	Hydrogen Bond	Carbon Hydrogen Bond
	SER407	3.5412	Hydrogen Bond	Carbon Hydrogen Bond
	SER407	3.15131	Hydrogen Bond	Carbon Hydrogen Bond
	TYR415	5.6983	Other	Pi-Sulfur
**9-2IJN**	TYR415	2.58825	Hydrogen Bond	Conventional Hydrogen Bond
	TYR448	2.60193	Hydrogen Bond	Conventional Hydrogen Bond
	SER556	2.39129	Hydrogen Bond	Conventional Hydrogen Bond
	ASP318	2.30386	Hydrogen Bond	Conventional Hydrogen Bond
	SER556	3.00339	Hydrogen Bond	Pi-Donor Hydrogen Bond
	MET414	4.45235	Hydrophobic	Alkyl
	CYS366	4.05039	Hydrophobic	Pi-Alkyl
**10-2IJN**	ASP225	4.67678	Electrostatic	Attractive Charge
	ASP318	3.98083	Electrostatic	Attractive Charge
	ASN291	2.27352	Hydrogen Bond	Conventional Hydrogen Bond
	ASP318	2.48923	Hydrogen Bond	Conventional Hydrogen Bond
	ASP318	3.36036	Hydrogen Bond	Conventional Hydrogen Bond

	ASN316	3.44406	Hydrogen Bond	Carbon Hydrogen Bond
	ASP318	3.37181	Electrostatic	Pi-Anion
	PHE193	5.848	Other	Pi-Sulfur
	VAL52	5.16073	Hydrophobic	Pi-Alkyl
	ARG158	5.23469	Hydrophobic	Pi-Alkyl
	CYS223	5.19777	Hydrophobic	Pi-Alkyl
**Reference (221)-2IJN**	ASN316	2.88031	Hydrogen Bond; Halogen	Conventional Hydrogen Bond; Halogen (Fluorine)
	CYS366	2.85929	Hydrogen Bond; Halogen	Conventional Hydrogen Bond; Halogen (Fluorine)
	TYR415	2.63377	Hydrogen Bond; Halogen	Conventional Hydrogen Bond; Halogen (Fluorine)
	TYR448	2.5187	Hydrogen Bond	Conventional Hydrogen Bond
	GLY449	2.18759	Hydrogen Bond	Conventional Hydrogen Bond
	ASN411	3.74024	Hydrogen Bond	Conventional Hydrogen Bond
	CYS366	4.37746	Hydrophobic	Alkyl
	LEU384	5.14024	Hydrophobic	Alkyl
	MET414	4.63661	Hydrophobic	Alkyl
	PHE193	5.20286	Hydrophobic	Pi-Alkyl
	TYR415	5.16217	Hydrophobic	Pi-Alkyl
	TYR448	4.87649	Hydrophobic	Pi-Alkyl
	CYS366	5.13737	Hydrophobic	Pi-Alkyl
	MET414	5.39688	Hydrophobic	Pi-Alkyl

Table S4. The optimized structures of the studied isothiazole derivatives.

No	Structure	No	Structure	No	Structure	No	Structure
**M1**	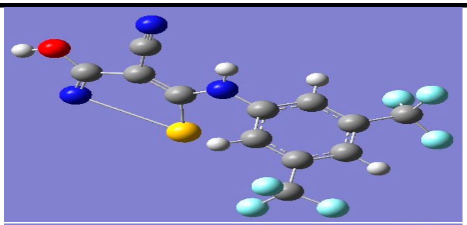	**M11**	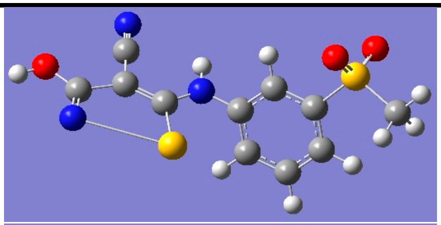	**M21**	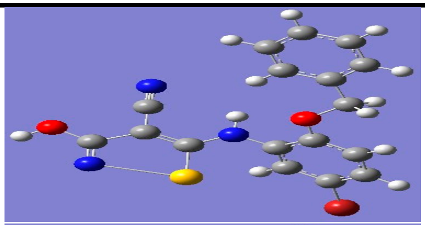	**M31**	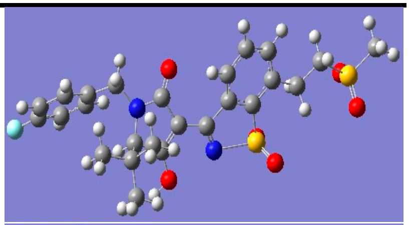
**M2**	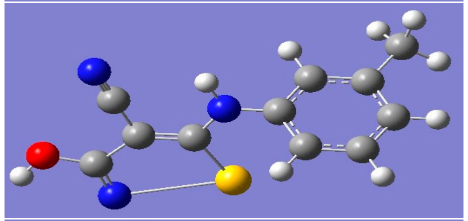	**M12**	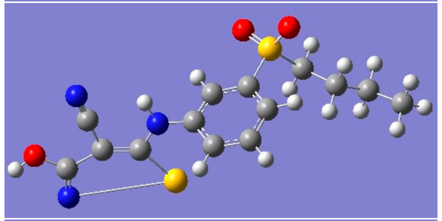	**M22**	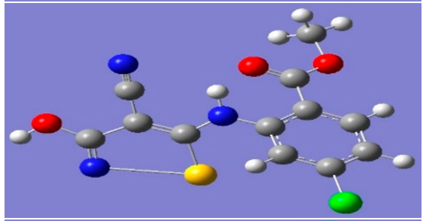	**M32**	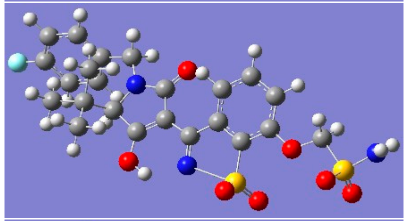
**M3**	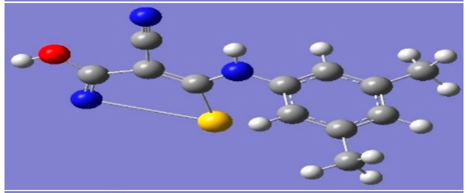	**M13**	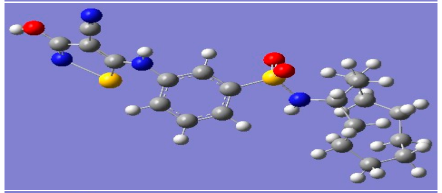	**M23**	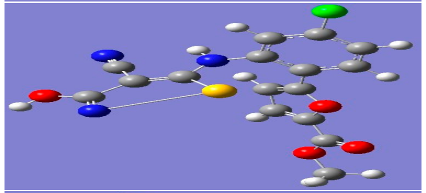	**M33**	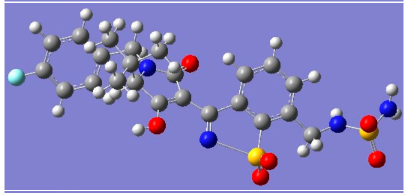
**M4**	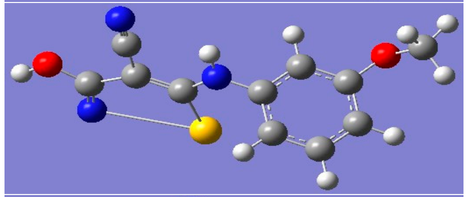	**M14**	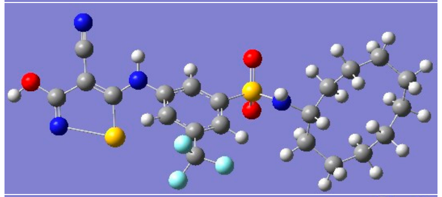	**M24**	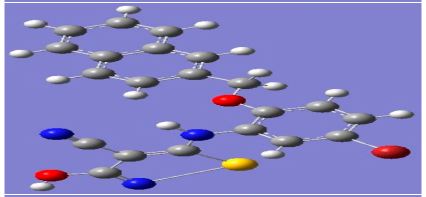	**M34**	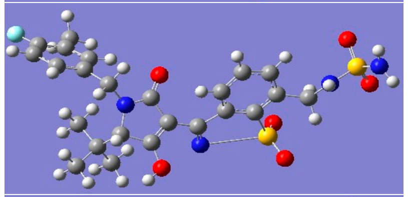
**M5**	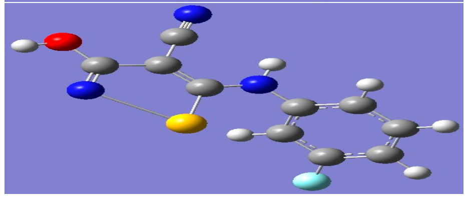	**M15**	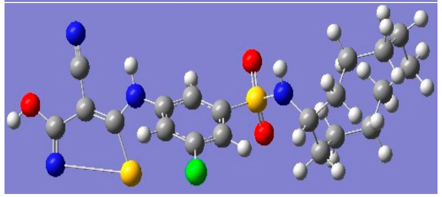	**M25**	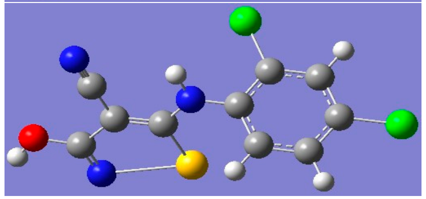	**M35**	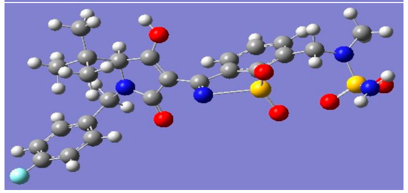

M6
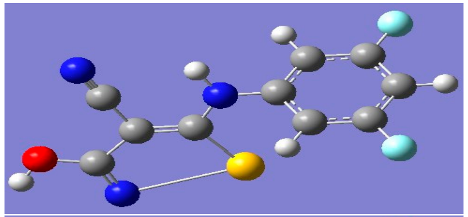

M7
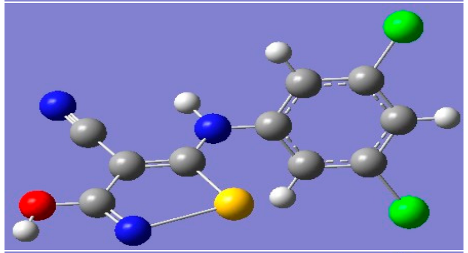

M8
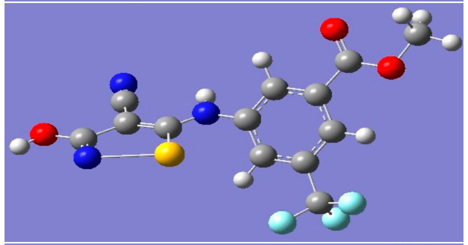

M9
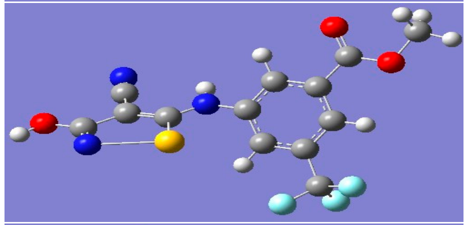

M10
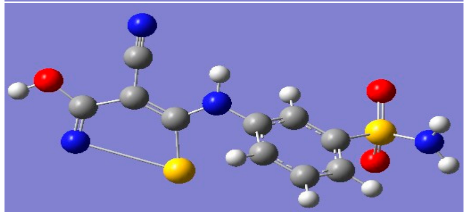

M16
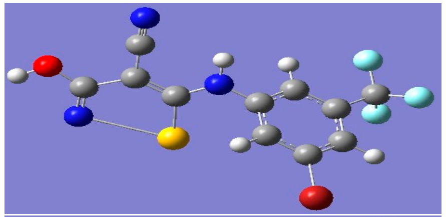

M17
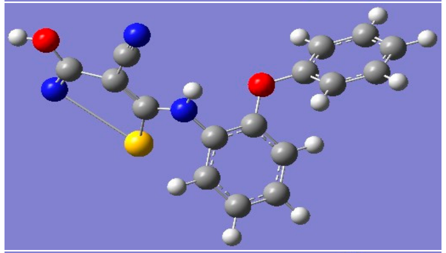

M18
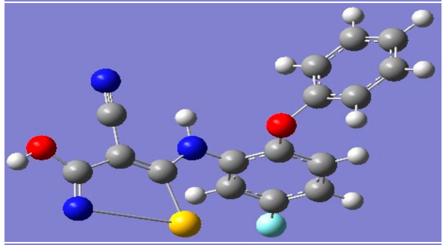

M19
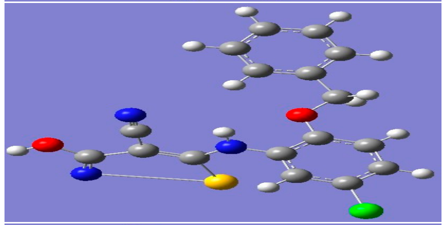

M20
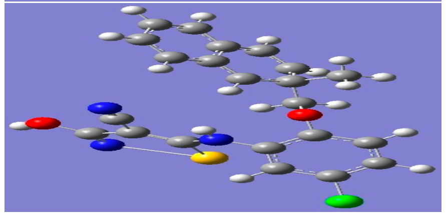

M26
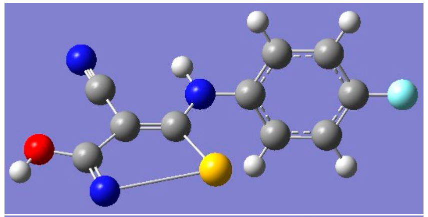

M27
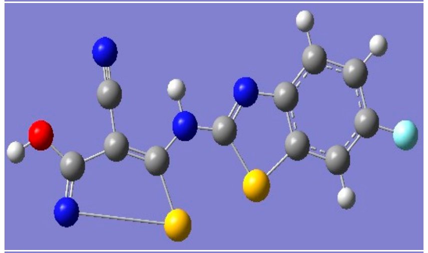

M28
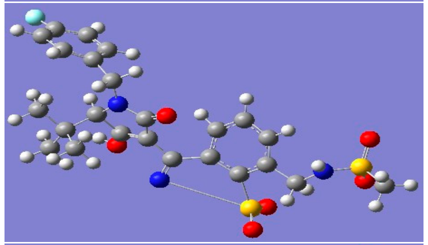

M29
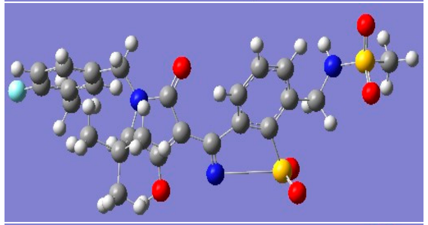

M30
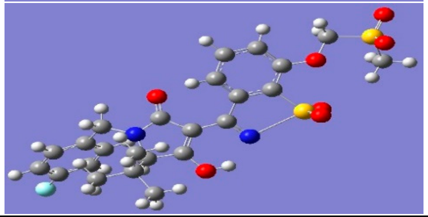

M36
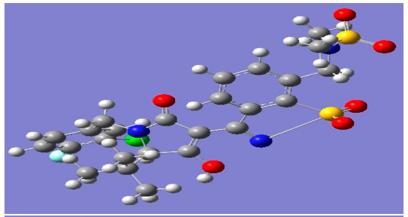

M37
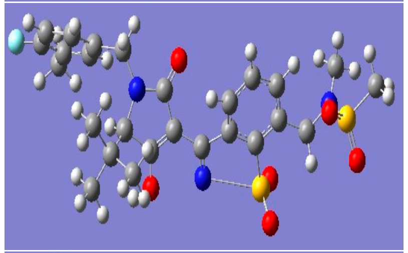

M38
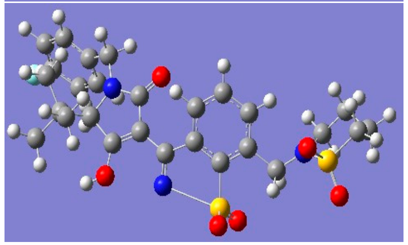

Table S5. The five criteria employed to assess the predictive capability of the model as outlined by Tropsha and Golbraikh pertain to the external validation set.

	Criteria			Note
**1**	$Q^2_{Loo} > 0.5$			Q^2_{Loo} is the leave-one-out cross-validation coefficient.
**2**	$R^2_{pred} = 1 - \frac{\sum(Yexp - Ypred)^2}{\sum(Yexp - \overline{Y}(train))^2} > 0.6$			R^2 is the determination coefficient between the experimental and predicted pIC50 values of test set compounds.<br>Ypred is the predicted activity of the training set compounds.<br>$\overline{Y}$(train) is the mean observed activity of the training set compounds.
**3**	$\frac{(R^2 - R_0^2)}{R^2} < 0.1$	or	$\frac{(R^2 - R'^2_0)}{R^2} < 0.1$	R_0^2 and $R'_0{}^2$ are the determination coefficients of predicted versus experimental and experimental versus predicted biological activity values.
**4**	$0.85 \leq K \leq 1.15$	or	$0.85 \leq K' \leq 1.15$	K and K′ are the slopes of regression lines through the origin for fits to experimental and predicted data, respectively.
**5**	$\lvert R_0^2 - R'^2_0 \rvert < 0.3$			R_0^2 is the squared correlation coefficient between observed and predicted values, and $R'_0{}^2$ is the squared correlation coefficient between observed and predicted values with the intercept value set to zero.
**6**	$r^2_m = R^2(1 - \sqrt{\lvert R^2 - R_0^2 \rvert}$			Matrix for external validation.
